# The T cell receptor sequence influences the likelihood of T cell memory formation

**DOI:** 10.1016/j.celrep.2024.115098

**Published:** 2024-12-27

**Authors:** Kaitlyn A. Lagattuta, Ayano C. Kohlgruber, Nouran S. Abdelfattah, Aparna Nathan, Laurie Rumker, Michael E. Birnbaum, Stephen J. Elledge, Soumya Raychaudhuri

**Affiliations:** 1 Center for Data Sciences, Brigham and Women’s Hospital, Boston, MA, USA; 2 Division of Genetics, Department of Medicine, Brigham and Women’s Hospital and Harvard Medical School, Boston, MA, USA; 3 Department of Biomedical Informatics, Harvard Medical School, Boston, MA, USA; 4 Program in Medical and Population Genetics, Broad Institute of MIT and Harvard, Cambridge, MA, USA; 5 Division of Rheumatology, Inflammation, and Immunity, Department of Medicine, Brigham and Women’s Hospital and Harvard Medical School, Boston, MA, USA; 6 Department of Genetics, Harvard Medical School, Boston, MA, USA; 7 Division of Immunology, Boston Children’s Hospital, Boston, MA, USA; 8 Koch Institute for Integrative Cancer Research, Cambridge, MA, USA; 9 Department of Biomedical Engineering, Massachusetts Institute of Technology, Cambridge, MA, USA; 10 Ragon Institute of MGH, MIT, and Harvard, Cambridge, MA, USA; 11 Howard Hughes Medical Institute, Chevy Chase, MD, USA; 12 Lead contact

## Abstract

The amino acid sequence of the T cell receptor (TCR) varies between T cells of an individual’s immune system. Particular TCR residues nearly guarantee mucosal-associated invariant T (MAIT) and natural killer T (NKT) cell transcriptional fates. To define how the TCR sequence affects T cell fates, we analyze the paired αβTCR sequence and transcriptome of 961,531 single cells. We find that hydrophobic complementarity-determining region (CDR)3 residues promote regulatory T cell fates in both the CD8 and CD4 lineages. Most strikingly, we find a set of TCR sequence features that promote the T cell transition from naive to memory. We quantify the extent of these features through our TCR scoring function “TCR-mem.” Using TCR transduction experiments, we demonstrate that increased TCR-mem promotes T cell activation, even among T cells that recognize the same antigen. Our results reveal a common set of TCR sequence features that enable T cell activation and immunological memory.

## In brief

The human immune system contains a vast repertoire of somatically recombined T cell receptor (TCR) sequences. To connect TCR sequence to T cell function, Lagattuta et al. analyze the paired αβTCR and transcriptome of >960,000 T cells. They identify TCR amino acids that promote T cell activation and immunological memory.

## Graphical Abstract

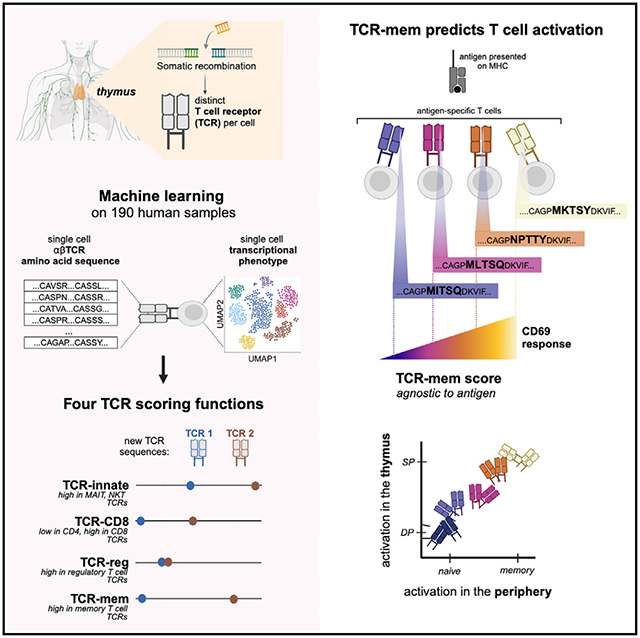

## INTRODUCTION

T cells are critical lymphocytes of the adaptive immune system. Early in T cell development, stochastic genome rearrangement on chromosomes 7 and 14 defines each T cell with its own T cell receptor (TCR).^1^ In the thymus and periphery, T cell differentiation depends critically on TCR activation.^2–8^ One prominent example is the *PLZF*^high^, innate-like transcriptional fate, which is nearly guaranteed when V(D)J recombination selects *TRAV1–2* and *TRAJ33*, *TRAJ20*, or *TRAJ12*.^9^

Given the central role of the TCR in T cell activation and differentiation, we^10^ and others^11–16^ have identified differences in TCR sequences (e.g., hydrophobicity, V gene selection) using bulk sequencing of flow-sorted T cell populations. However, bulk sequencing obscures the pairing of TCR α and β chains. Furthermore, flow sorting requires predefining T cell states for investigation and may miss important transcriptional heterogeneity of T cells.

Now, single-cell sequencing assays enable joint profiling of the TCR and transcriptome. In contrast to bulk sequencing methods, single-cell technology anchors α and β TCR reads to individual cells, allowing reconstruction of the full TCR α/β heterodimer. Moreover, genome-wide transcriptional analysis can comprehensively define T cell states. Early methods to jointly analyze TCR and transcriptional data^17,18^ have suggested that TCR sequence similarity may correspond to similarity in transcriptional state.

To statistically define the relationship between the TCR sequence and T cell state, we analyze 961,531 T cells with quality-controlled αβTCR and transcriptional profiling at single-cell resolution from seven published datasets (Table 1). Rather than pre-specifying transcriptional states, we use paired dimensionality reduction to uncover relevant transcriptional states in an unbiased fashion. Our results define four TCR scoring functions that quantify the transcriptional fate predisposition conferred by the TCR. We apply these scoring functions to better understand thymic selection as well as cell state variation within antigen-specific T cell populations.

## RESULTS

### T cell transcriptional state annotation

**Figure 1.**
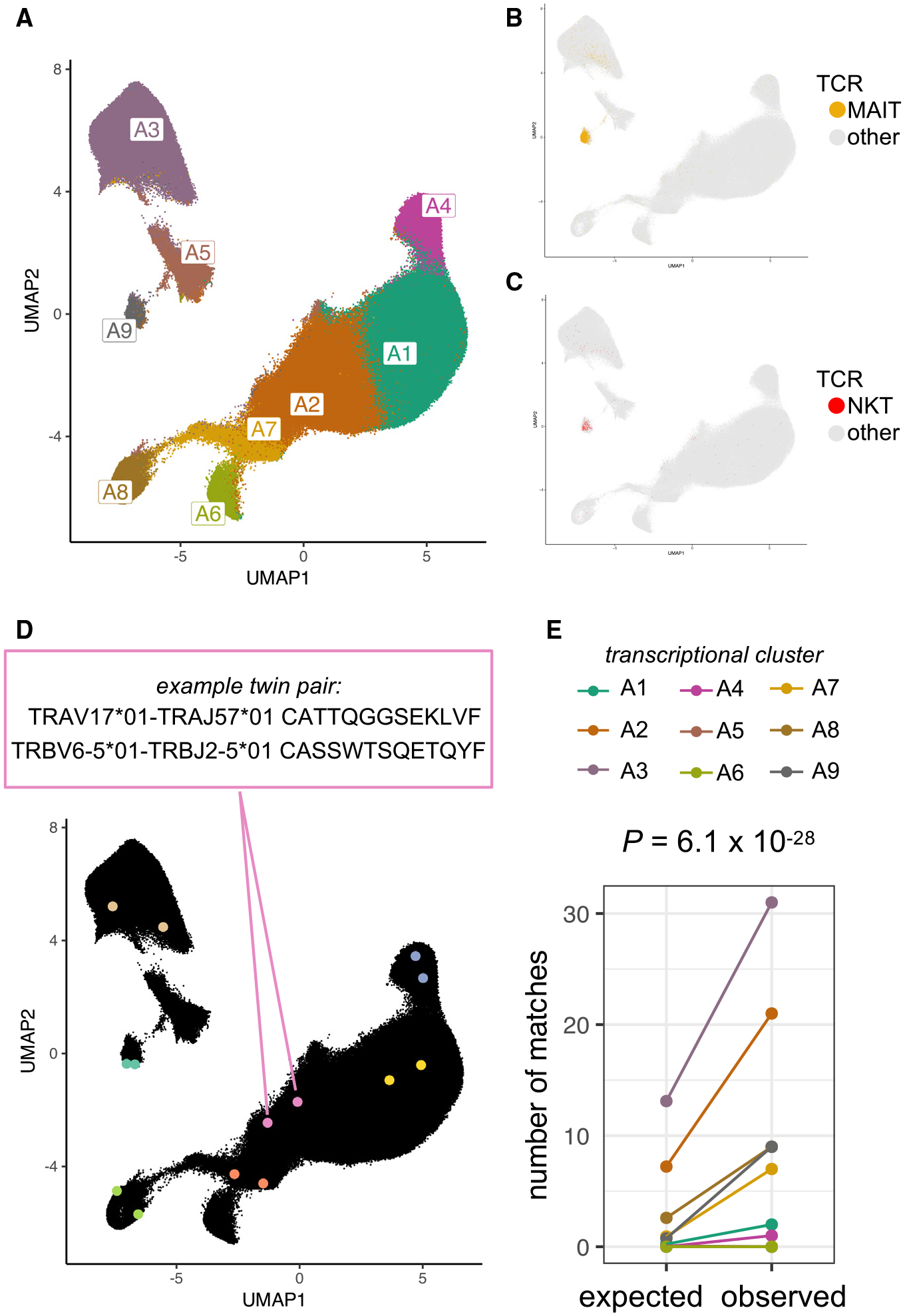
T cells with matching TCR sequences reach similar transcriptional fates in different individuals (A) Uniform manifold approximation and projection (UMAP) of T cells from dataset 1 based on the first 20 batch-corrected principal components (PCs) of gene expression. Cells are colored by transcriptional cluster, named according to expression of marker genes and proteins (see Figure S1). (B and C) T cells from dataset 1, colored yellow (B) if their paired TCR sequence includes the canonical genes for MAIT cells (TRAV*1–2 with TRAJ*20, -*33, or -*12 and TRBV*6 or -*20) or colored red (C) if their paired TCR sequence includes the canonical genes for NKT cells (*TRAV10* with *TRAJ18* and *TRBV25–1*) or gray otherwise. (D) Seven example TCR-twin cell pairs from dataset 1 are highlighted in distinct colors. Within a twin pair, TCRs with matching sequences were observed in two different individuals. (E) Number of transcriptional cluster matches for TCR-twin cell pairs in each cluster. Real observed counts are compared to expected counts based on random sampling of T cells without regard for TCR sequence. *p* value is computed by binomial exact test with *n* = 115 TCR-twin pairs.

To construct an accurately annotated reference of T cells, we used dataset 1 (from the COMBAT consortium^19^; Table 1) with 371,621 T cells from 122 individuals with multimodal TotalSeq profiling of mRNA and surface proteins. We clustered the cells in two ways: first, agnostic to protein expression (clusters A1–A9; Figures 1A and S1), and second, incorporating traditional surface markers via a linear multimodal strategy^26,27^ (clusters B1–B9; Figure S2). Cluster A9, representing the *PLZF*^high^ innate-like T cell state, accounted for nearly all canonical mucosal-associated invariant T (MAIT) or natural killer T (NKT) TCRs (Figures 1B and 1C). Clusters B1–B9 delineated CD4, CD8, central memory (CM), and effector memory (EM) states based on a curated list of 10 surface proteins, including CD45RO and CD45RA (Figure S2; STAR Methods). To standardize cell state definitions across datasets, we projected all additional datasets (Table 1) into these two embeddings and transferred annotations via k-nearest-neighbors classification (k = 5; STAR Methods).

### Transcriptional fate matching between T cells with the same TCR sequence from different individuals

We first used dataset 1 (Table 1) to assess whether perfect TCR sequence matching raised the likelihood of T cell state matching. Within an individual, identical TCR sequences likely reflect an expanded T cell clone, comprised of cells that tend to be transcriptionally similar. To avoid clonally related cells, we focused on identical TCR sequences sampled from two different individuals. We identified 115 pairs of “TCR twins”: two T cells from two different individuals with the same TCR α and β amino acid sequences (Figure 1D; STAR Methods). To test the general relationship between the TCR sequence and T cell state, we asked whether the transcriptional states of TCR twins were concordant more often than expected by chance. For statistical power in this analysis, we used relatively coarse clusters to define transcriptional states (A1–A9; Figure 1A).

Under the null, we would expect 25 (of 115) transcriptionally concordant TCR-twin pairs (see STAR Methods). Instead, we observed 80 (*p* = 6.1 × 10^−28^, exact binomial test, Figure 1E; STAR Methods). To assess if this enrichment was explained by major histocompatibility complex (MHC)(-like) restriction, we repeated our analysis with MAIT cell and NKT cell TCRs removed and observed an enrichment in concordant states (*p* = 2.3 × 10^−21^, exact binomial test). Partitioning into CD4^+^ and CD8^+^ populations did not obviate enrichment either (*p* = 4.0 × 10^−9^, *p* = 0.00018, exact binomial test). Recognizing that SARS-CoV-2 infection is a potential confound, we filtered to individuals that were PCR negative for SARS-CoV-2 but continued to observe enrichment in both dataset 1 and dataset 2 (13 matches versus 6 expected by chance, *p* = 0.00048, exact binomial test; 11 matches versus 6 expected by chance, *p* = 0.00138, exact binomial test). To account for shared human leukocyte antigen (HLA) alleles, we repeated this analysis in an HLA-genotyped dataset^25^ and continued to observe enrichment regardless of whether the TCR twins were sampled from individuals with a shared HLA allele (*p* = 2.9 × 10^−101^, exact binomial test). Indeed, neither matched SARS-CoV-2 status nor matched HLA allele(s) significantly increased TCR twinning or similarity. These results suggest a consistent influence of TCR sequence on T cell fate in unrelated individuals.

This analysis is limited to TCRs found in more than one individual (“public TCRs”), which comprise <0.01% of the TCRs in this cohort. Public TCRs have distinct structural features^28^ and could demonstrate transcriptional state matching due to recognition of common antigens. We hypothesized, however, that these results were driven to some extent by the distinct biophysical features of the TCR sequences. If so, similar, but not identical, TCR sequences would also promote similar T cell fates.

### A multidimensional approach to uncover TCR sequence features that guide T cell fate

**Figure 2.**
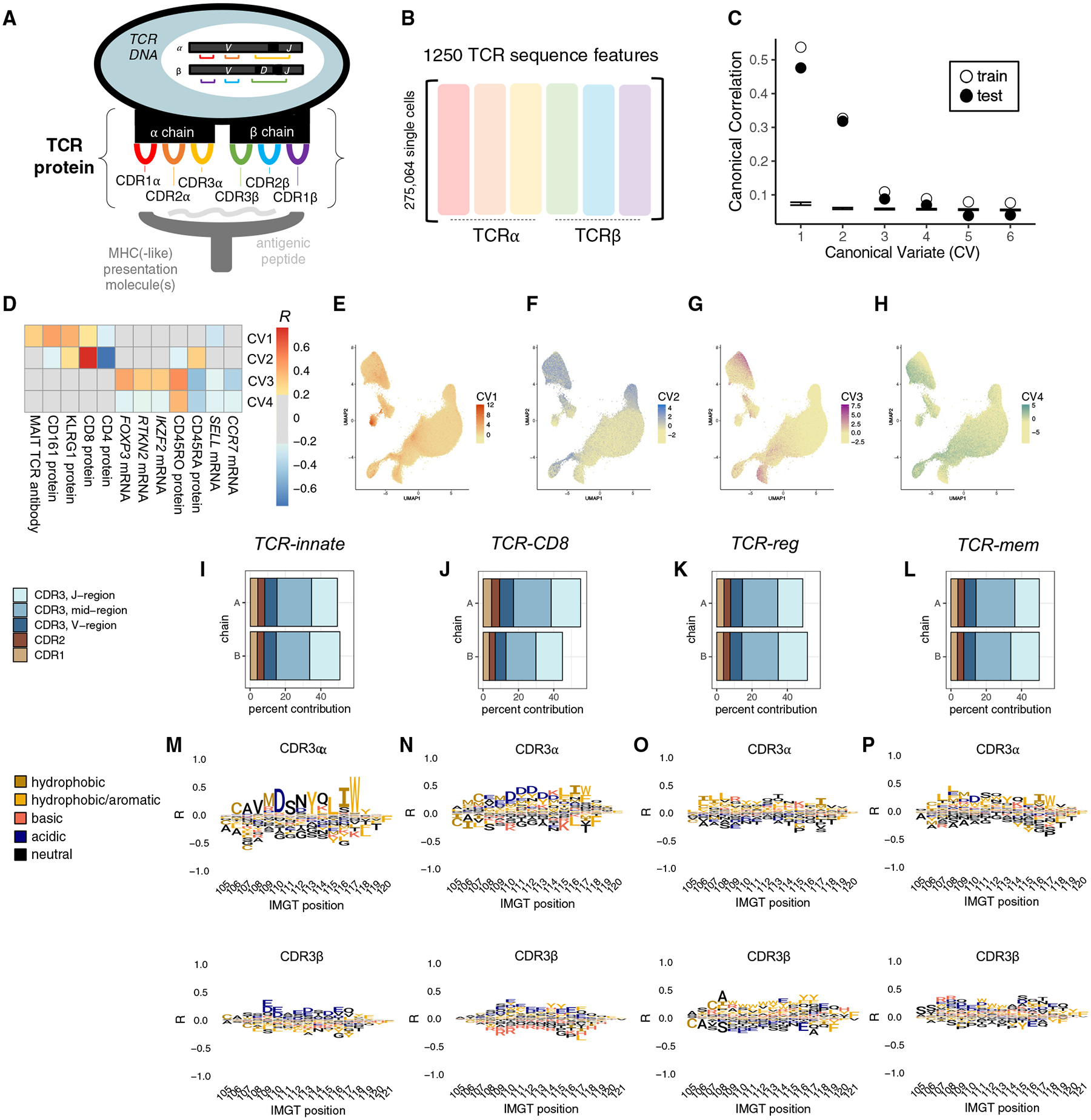
A quantitative approach to uncover TCR sequence features that influence T cell fate Schematic of a T cell recognizing antigen through the T cell receptor (TCR), an αβ heterodimeric surface protein encoded by V, D, and J genes that are stochastically rearranged in each T cell. Black regions in between V, D, and J genes represent non-templated nucleotide insertions. The TCR surface protein contains six complementarity-determining regions (CDRs) that protrude toward the presented antigen: CDR1α, CDR2α, CDR3α, CDR1β, CDR2β, and CDR3β. (B) Our quantitative representation of TCR sequence data. We extract 1,250 sequence features from each TCR sequence, representing hydrophobicity, size, charge, secondary structure, and heat capacity of the amino acids in the six CDRs. (C) Canonical correlations between TCR and T cell state detected in training and held-out testing observations. Error bars denote the minimum and maximum canonical correlations observed in 1,000 permutations of the data. (D) Heatmap depicting Pearson correlation between genes and proteins in dataset 1 and the continuous T cell state identified by each canonical variate (CV). (E–H) UMAP of dataset 1 T cells, colored by T cell state scores for CV1, CV2, CV3, and CV4, respectively. (I–L) Percentage of contribution from each TCR region toward the TCR scoring function. (M–P) CDR3 amino acid contributions to each TCR scoring function, visualized as marginal correlations to the TCR score. Contributions from other TCR regions are displayed in Figure S4.

To extend our study to private TCRs, we converted each TCR sequence into a vector of biophysical features. Consistent with other numerical representations of the TCR,^17,29^ we translated each amino acid residue in both the α and β chains of the TCR into five Atchley factors.^30^ These factors correspond to hydrophobicity, size, charge, secondary structure, and heat capacity. Applying this to α and β complementarity-determining and framework regions of the TCR yielded 1190 biophysical features. Excluding invariant positions and framework regions and adding interaction terms between adjacent residues yielded 1,250 TCR features (Figures 2A and 2B; Table S2; STAR Methods).

We aimed to identify TCR sequence effects on T cell state in an unbiased fashion, without restricting to preselected cell states. To find generalizable associations, we combined dataset 1 with a second published dataset (dataset 2, Table 1; STAR Methods), resulting in 494,419 quality-controlled T cells from 256 individuals. We applied regularized canonical correlation analysis (rCCA) (STAR Methods). Each axis identified by rCCA denotes a weighted sum of gene expression principal component (PC) scores (a transcriptional state) that correlates with a weighted sum of TCR sequence features.

We mitigated technical confounding in our rCCA implementation. First, to prevent confounding by variable clone size, we selected one cell at random to represent each clone. We confirmed that results were invariant to the specific set of cells sampled (STAR Methods). To mitigate overfitting, we added a ridge regularization to the covariance matrix for each of the inputs to rCCA, using 5-fold cross-validation to tune both lambda penalty values. To assess overfitting, we randomly assigned 68 donors to a validation test set, such that ~30% of clones (29.3%) were held out from training.

### rCCA identifies four T cell fates informed by TCR sequence

We observed canonical correlations between the TCR sequence and T cell state descending from *R* = 0.54. To assess the statistical significance of each canonical correlation, we permuted our data 1,000 times and re-applied rCCA (STAR Methods). We observed empirical *p* values <0.001 for both training and held-out testing data for the first four canonical variates (CVs) (Figure 2C).

To interpret the four continuous T cell states identified by rCCA, we examined CVs 1–4 in terms of cell scores and expression correlates (Figures 2D–2H; Table S3; STAR Methods). Cells scoring the highest on CV1 localized to transcriptional cluster A9 (Figures 2E and S3A), the innate-like, *PLZF*^high^ transcriptional fate for canonical MAIT and NKT cell TCRs (Figures 1B and 1C). CV2 tracked closely with CD8 versus CD4 surface expression, delineating CD4^+^ T versus CD8^+^ T populations (Figures 2F and S3B–S3D). These results point to families of peptide presentation molecules as the primary source of covariation between the TCR sequence and T cell state. Indeed, it is well established that unconventional (MR1, CD1d), MHC class I, and MHC class II families each prefer biophysically distinct αβTCR sequences.^9,13,14,31–35^

In addition to these known relationships, rCCA proposed previously unknown connections between the TCR sequence and T cell state. CV3 highlighted TCR sequence similarity between *FOXP3*-expressing CD4^+^ regulatory T (T_reg_) cells and KIR+*HELIOS*+ CD8 T cells (Figures 2G and S3E–S3K), which have recently been described as human CD8^+^ T_reg_ cells.^36^ This suggests that the same TCR sequence features may promote suppressive functional states in both the CD4 and CD8 compartments. Most strikingly, CV4 appeared to capture TCR sequence differences between naive and memory T cells (Figures 2H and S3L). Surface protein measurements indicated that both EM and CM CD4^+^ and CD8^+^ T cells scored highly on CV4 (Figures S3M and S3N). This raises the intriguing possibility that some sequence features render the TCR more generally prone to activation.

### TCR scoring functions quantify TCR sequence features that inform T cell fate

The continuous T cell states defined by rCCA nominated four contrasts in T cell state for further study: *PLZF*^high^ versus other, CD8T versus CD4T, regulatory T (T_reg_) versus conventional T (T_conv_), and memory versus naive. For each of these recognizable T cell fate decisions, we used logistic regression on the same observations from datasets 1 and 2 to learn a more precise predictive weighting scheme on the 1,250 TCR sequence features (Table S2; STAR Methods). As a secondary analysis, we trained separately on COVID-positive and COVID-negative samples and verified that these predictive weighting schemes were robust to infection status (Figures S4A and S4B). We named each predictive weighting scheme, or TCR scoring function, by the T cell state of interest: “TCR-innate”’ to predict the innate-like, *PLZF*^high^ state, “TCR-CD8” to predict the CD8^+^ state, “TCR-reg” to predict the T_reg_ cell state, and “TCR-mem” to predict the memory state.

To interpret each of these TCR scoring functions, we examined relative contributions from each complementarity-determining region (CDR) and amino acid residue (Figures 2I–2P and S4C–S4F; STAR Methods). TCR-innate was characterized by critical amino acids in CDR2α, reflecting *TRAV* gene selection (Figure S4C). As expected from previous studies,^13,14^ TCR-CD8-high sequences were depleted for positive charge in the junctional mid-region of CDR3 (Figure 2N). TCR-reg reflected increased hydrophobic CDR3β residues in CD4 T_reg_ cells, consistent with previous reports.^10,12^ Paired αβ TCR sequencing data revealed that enrichment for hydrophobic amino acids extended to CDR3α (Figure 2O). For TCR-mem, feature dependence analysis highlighted the importance of CDR3α and CDR3β (Figure 2L). We assessed TCR-reg and TCR-mem separately in CD4^+^ and CD8^+^ T cells and observed that these TCR scoring functions were equally applicable to both lineages (Figures S4G and S4H; STAR Methods).

### TCR scoring functions generalize across individuals

**Figure 3.**
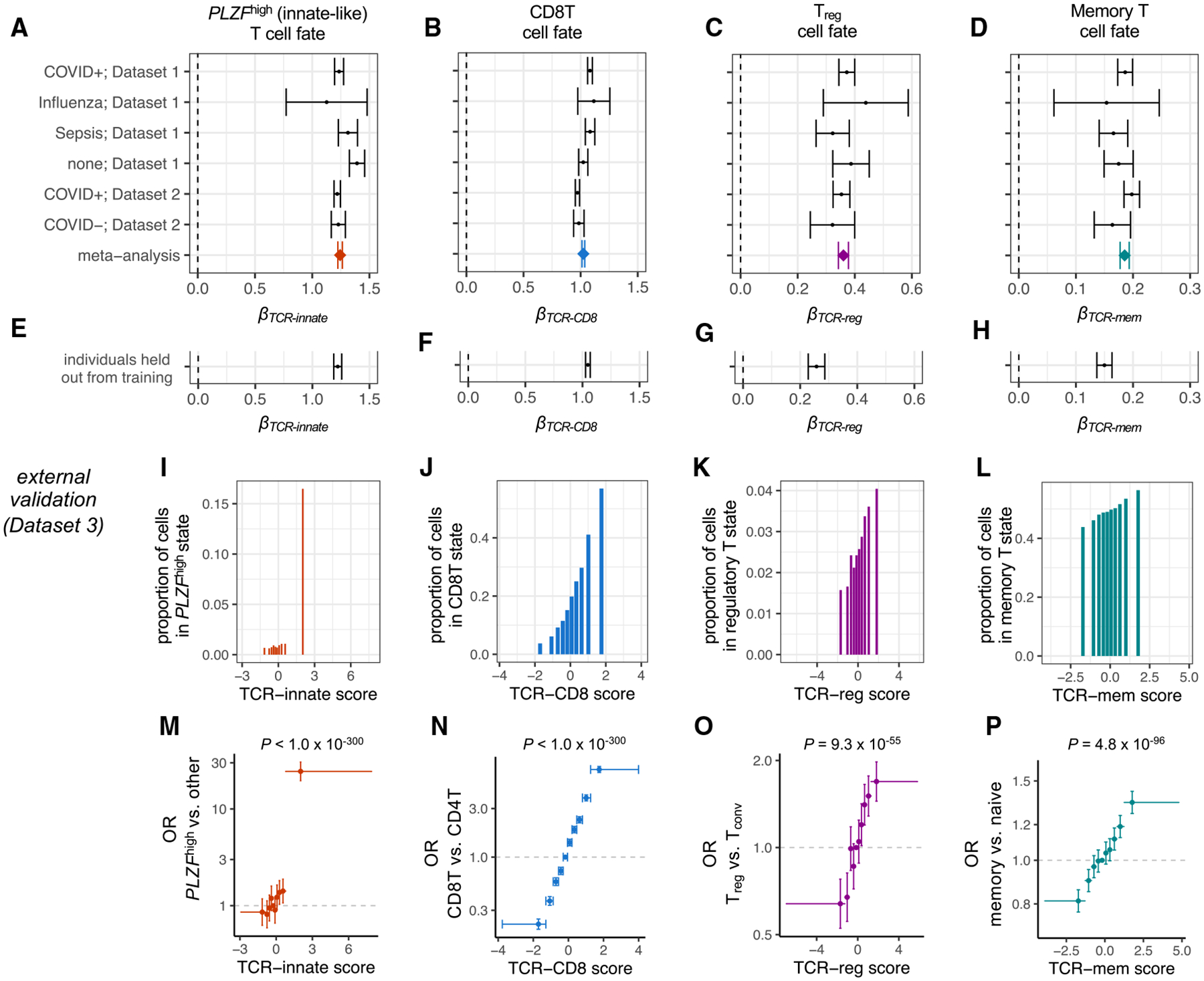
TCR scoring functions generalize across individuals (A) Forest plot depicting the association between TCR-innate and *PLZF*^high^ T cell state for T cells from each subset of individuals. *β*_*TCR-innate*_ is computed via mixed-effects logistic regression, with TCR-innate scaled to mean 0, variance 1. Error bars denote 95% confidence intervals. Meta-analytic *β*_*TCR-innate*_ is estimated by fixed-effects inverse-variance-weighted meta-analysis. (B–D) Remaining forest plots depict the same association tests for (B) TCR-CD8 and CD8T cell state, (C) TCR-reg and T_reg_ cell state, and (D) TCR-mem and memory T cell state. (E–H) 95% confidence intervals for *β*_*TCR-innate*_, *β*_*TCR-innate*_, *β*_*TCR-innate*_, and *β*_*TCR-innate*_, respectively, in the individuals from dataset 1 and dataset 2 held out from TCR score training. (I–L) Proportion of T cell clones from an external validation dataset (dataset 3) observed in each T cell state of interest within each decile of its corresponding TCR score. (M) Each point represents a decile of the TCR-innate score in dataset 3, with a horizontal bar spanning from its minimum to maximum value. We compute the odds ratio (OR; y axis) for the *PLZF*^high^ T cell state for T cells in each decile compared to T cells in the fifth decile. 95% CIs (error bars) and *p* values were computed via mixed-effects logistic regression. (N–P) Dataset 3 T cells as in (M) but for CD8T, T_reg_, and memory T cell states and their corresponding TCR scores. For (A)–(H), data are represented as log OR ± 95% confidence interval. For (M)–(P), data are represented as OR ± 95% confidence interval. *p* values were computed by the Wald test through mixed-effects logistic regression, *n* = 106,129 T cells.

We considered the possibility that the associations between the TCR sequence and T cell state were driven by a subset of individuals. We first stratified each dataset by clinical status (COVID, sepsis, influenza, none of the above) and used mixed-effects logistic regression to calculate *β*_*TCRscore,*_ the association between the TCR score calculated based on both α and β chains, and the target T cell state (*β*_*TCRscore*_ = log odds ratio [OR] per standard deviation increase in TCR score; see STAR Methods). In each clinical stratum, we observed a statistically significant positive association for each TCR scoring function (24 tests, maximum *p* = 0.001; Figures 3A–3D; Table S4). Reassured that our TCR scoring functions were not driven by the clinical subset, we considered the possibility of an unknown individual-level mediator, such as HLA genotype. We computed *β*_*TCR-innate*_, *β*_*TCR-CD8*_, *β*_*TCR-reg,*_ and *β*_*TCR-mem*_ within each individual’s T cells separately and estimated the proportion of individuals for whom our TCR score does not raise the odds of its target T cell state(the local false sign rate^37^). Random effects meta-analysis indicated a near-zero proportion for each of our four TCR scoring functions (<1 × 10^−6^; Figures S4I–S4L; STAR Methods). We concluded that since our TCR scoring functions were robust to inter-individual variation, they should generalize to unseen samples.

We next applied our TCR scoring functions to data outside of our training set. In the 30% of dataset 1 and dataset 2 T cell clones held out from training, we observed replication of each TCR scoring function (Figures 3E–3H; STAR Methods). In an external dataset of peripheral blood T cells (dataset 3, Table 1), we observed a consistent and statistically significant increase in the odds of the T cell state of interest for each TCR scoring function (Figures 3I–3P; Table S4). Compared to T cells in the lowest TCR-mem decile, T cells in the highest TCR-mem decile had a 66% greater odds of being observed in a memory state (OR = 1.66, 95% confidence interval [CI] = [1.57–1.76], *p* = 3.5 × 10^−68^; Table S4). These results indicate a substantial role for TCR-mem in shaping the odds of T cell memory formation.

### Alternative TCR scoring schemes

We next benchmarked our TCR scoring functions against existing TCR metrics. We previously developed a T_reg_ cell TCR scoring function, “TiRP,” using the TCR β chain alone.^10^ With the additional information of the α chain, TCR-reg clearly outperformed TiRP (*β*_*TiRP*_ = 0.14, 95% CI = [0.10–0.18]; *β*_*TCR-reg*_ = 0.29, 95% CI = [0.25–0.32]; dataset 3 CD4T cells). Amino acid interaction strength^38^ (AAIS) has been postulated to estimate a TCR’s average affinity to peptide-MHC (pMHC),^39^ but this has not been directly tested. In dataset 3, increasing AAIS corresponded to an increase in the odds of memory state only when applied to CDR3 amino acids (*β*_*AAIS*_ = 0.02, 95% CI = [0.003–0.03], *p* = 0.008). The effect size for AAIS was minimal compared to TCR-mem (*β*_*TCR-mem*_ = 0.14, 95% CI = [0.12–0.15]), however. Including AAIS as a covariate did not substantially change the estimated effect size of TCR-mem (conditional *β*_*TCR-mem*_ = 0.14, 95% CI = [0.12–0.15], heterogeneity *p* = 0.92). TCR-reg and TCR-mem clearly outperform these alternative TCR scoring functions by capturing both α and β TCR sequence features that promote recognition in the context of the TCR-pMHC interface.

We next wanted to assess if more complex models would provide better TCR scoring functions. For each of the four T cell states of interest, we trained a convolutional neural network (CNN) to detect possibly nonlinear associations between TCR amino acid motifs and T cell fate (STAR Methods). However, this deep learning approach provided no substantial benefit in discovery or external validation data (Figure S5; Table S6).

### Untranslated products of V(D)J recombination do not affect T cell fate

Because stochastic V(D)J recombination precedes T cell fate decisions, TCR sequence associations to T cell state likely reflect causal effects of V(D)J recombination. However, a causal pathway that begins with V(D)J recombination and ends with the T cell state likely includes several important biological mediators. To better understand these mediators, we decomposed V(D)J recombination into three products: (1) DNA-level excisions and insertions, (2) amino acid changes in the surface TCR, and (3) antigen recognition. To isolate (1) from (2), we analyzed nonproductive V(D)J recombination sequences that are not translated into surface TCR proteins. Then, to distinguish (3) from (2), we examined the TCR sequences and T cell states of antigen-labeled single cells.

Nonproductive TCR sequence transcripts can be detected when an out-of-frame V(D)J recombination event on one chromosome is followed by an in-frame V(D)J recombination event on the other.^40^ Due to stop codons and frameshift errors, these nonproductive TCRs represent V(D)J genome rearrangements that are not translated into surface antigen receptors.^41^ To assess whether these DNA-level changes are sufficient to produce the observed effects on T cell state, we applied our TCR scoring functions to nonproductive TCR sequences (dataset 4, Table 1). We observed no evidence of association for any of the four TCR scoring functions (*p* > 0.05). Down-sampling did not obviate associations for productive TCRs, confirming that the lack of association was not due to reduced statistical power (Table S4; STAR Methods). We concluded that the DNA-level excisions and insertions from V(D)J recombination are, in general, not sufficient to affect T cell state; recombination products must be expressed at the protein level.

### TCR-mem predicts increased T cell activation within antigen-specific populations

**Figure 4.**
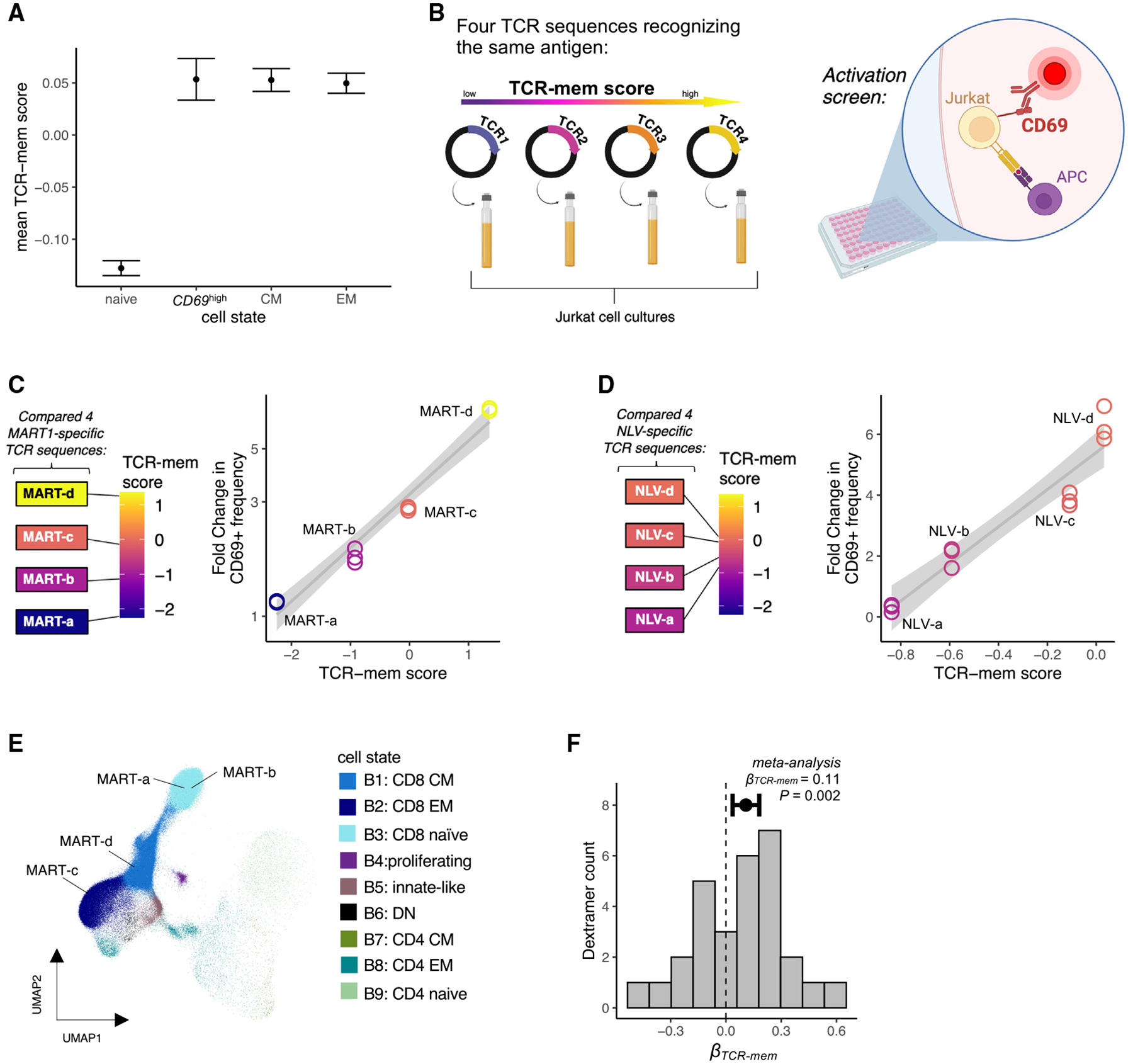
TCR sequences that recognize the same antigen can be poised for different fates (A) Mean TCR-mem scores of four mutually exclusive groups of T cells in dataset 1: naive (*CCR7*^high^CD45RA^+^CD45RO^−^), *CD69*^high^ (*CD69*^high^ *MK67*^high^CD38^+^HLA-DR^+^), central memory (CM; *CCR7*^low^*CD27*^high^CD45RA^−^CD45RO^+^), and effector memory (EM; *CCR7*^low^*CD27*^low^CD45RA^+^ for CD8s and CD45RO^+^ for CD4s). Data are represented as mean ± 95% confidence interval. (B) Schematic of our experimental validation using either MART-1 or NLV-reactive TCRs: TCR-knockout Jurkat cells were transduced with four antigen-reactive TCRs from the Dextramer dataset, co-cultured with antigen-presenting cells (APCs) expressing the antigen of interest, and stained for CD69 to screen for cellular activation. (C) For each MART-reactive TCR sequence, we compared its TCR-mem score (x axis) to the fold change in the frequency of CD69^+^ Jurkat cells (y axis) following exposure to antigen, compared to background CD69^+^ frequency in the absence of the MART-1 antigen. Measurements are in triplicate. (D) For each NLV-reactive TCR sequence, we compared its TCR-mem score (x axis) to the fold change in the frequency of CD69^+^ Jurkat cells (y axis) following exposure to antigen, compared to background CD69^+^ frequency in the absence of the NLV- antigen. Each measurement was done in triplicate. (E) T cells from Dextramer-labeled dataset (dataset 5) projected onto UMAP coordinates of a low-dimensional transcriptional space defined by dataset 1, with strong influence from Total-seq antibody counts for CD4, CD8, CD45RO, and CD45RA. Four T cells, with TCR sequences we refer to as MART-a, MART-b, MART-c, and MART-d, respectively, are labeled. (F) Histogram of 29 *β*_*TCR-mem*_ estimates for the 29 Dextramer-specific populations. Data are represented as log odds ratio ± 95% confidence interval. *p* value is computed by random effects meta-analysis, *n* = 29 *β*_*TCR-mem*_ estimates for the 29 Dextramer-specific populations.

T cell activation, marked by CD69 upregulation, is a critical first step in T cell memory formation.^42,43^ If TCR-mem increases the odds of memory formation by promoting early activation, then (1) recently activated T cells (*CD69*^high^, *MK67*^high^, CD38^+^, HLA-DR^+^) should exhibit high TCR-mem, and (2) this phenomenon should be apparent in Jurkat cells, which biologically resemble T cells in activation but not the subsequent steps of memory formation. We identified recently activated T cells (*CD69*^high^, *MK67*^high^, CD38^+^, HLA-DR^+^) in dataset 1 and observed that TCR-mem in this subset was just as high as TCR-mem in other memory subsets (Figure 4A). Thus, we hypothesized that TCR-mem sequence features promote T cell activation.

First, we tested whether higher TCR-mem led to greater cellular activation in Jurkat cells. We selected four naturally occurring TCR sequences found in human data^23^ that spanned the range of our TCR-mem metric and transduced each TCR into Jurkat cells (Figure 4B). All four TCR sequences recognize the same “ELA-” antigenic peptide from melanoma-associated antigen recognized by T cells (MART-1), presented on HLA-A*02:01.^23^ Thus, this array of TCR sequences allows us to examine whether changes in amino acid composition that increase TCR-mem indeed causally promote T cell memory formation while controlling for cognate antigen and HLA (Table 2).

Each TCR sequence exhibited greater CD69 upregulation compared to baseline (fold change > 1), confirming specific reactivity to the MART-1 antigen. However, the extent of activation, quantified by the percentage of CD69^+^ cells compared to baseline, clearly tracked with the TCR-mem score (Figure 4C). Thus, the TCR sequence features that we have observed to differ between memory and naive cells *in vivo* appear to causally promote T cell activation *in vitro*.

To assess the stability of this finding, we examined the CD69 response at different antigen doses. We titrated the amount of MART-1-expressing antigen-presenting cells (APCs) and repeated the overnight co-culture. These titrations showed the expected dose-response relationship, which indeed escalated with increasing TCR-mem (Figure S6A). At each antigen dose, increased TCR-mem scores corresponded to greater CD69 responses.

We next assessed if altering individual residues to increase or decrease TCR-mem tracked with changes in activation. For this, we examined a different antigen, the “NLV-” peptide from the cytomegalovirus (CMV) antigen pp65 presented on HLA-A*02:01. We repeated the co-culture experiment with four TCR sequences that differ by one to four amino acids in CDR3α but are otherwise identical in sequence (Table 2). These TCRs were synthetically engineered and previously shown to recognize the “NLV-” antigenic peptide (Schub et al.^44^ and Abdelfattah et al.^45^). As in the MART-1 experiments, we saw that each incremental gain in TCR-mem induced a greater fold change in activation (Figures 4D and S6B). Thus, our TCR-mem metric identifies replicable differences in immune reactivity between TCR sequences, even when sequence differences are as minor as one CDR3α amino acid.

To extend our analyses beyond these two HLA-A*02:01 antigens, we examined single-cell profiling data^23^ (dataset 5, Table 1) with 44 Dextramers, including HLA-A*02:01, HLA-B*07:02, and HLA-B*35:01. Briefly, 80 million CD8^+^ T cells from the peripheral blood of four human donors were exposed to 44 pMHC-bar-coded Dextramers. Multimodal sequencing then assayed the αβTCR sequence, transcriptome-wide expression, and pMHC Dextramer counts for each Dextramer-positive cell. Using Symphony and k-nearest neighbors, we assigned T cell states based on our multimodal T cell reference (Figure 4E).

Following custom normalization of Dextramer Unique Molecular Identifier (UMI) counts (Figures S7A–S7D; STAR Methods), we observed a mixture of transcriptional states within each antigen-specific population (Figure S7E). This transcriptional heterogeneity is consistent with other tetramer-sorted single-cell RNA sequencing (scRNA-seq) studies,^46–48^ demonstrating that T cells with the same antigen specificity vary in transcriptional phenotype. We wondered if TCR-mem helped to explain naive versus memory phenotypes within each antigen-specific population.

Within each antigen-specific population, we tested the association between TCR-mem and memory state using a single cell per TCR clone (STAR Methods). Consistent with our TCR transduction experiments, we observed *β* > 0 for the majority of antigens (19/29), including seven antigen-specific populations with a nominally significant (one-tailed *p* < 0.05) result (Table S7). For example, among T cells recognizing IPSINVHHY presented on HLA-B*35:01, memory T cells bore TCRs with significantly higher TCR-mem scores (logistic regression *β*_*TCR-mem*_ = 0.36, *p* = 0.02).

Given a lack of statistical power within each antigen-specific population (Figure S7F), we conducted a meta-analysis across antigen-specific populations. We observed a significant effect of TCR-mem on memory state, adjusted for antigen specificity (Figures 4F and S7G, logistic regression *β*_*TCR-mem*_ = 0.11, *p* = 0.002; STAR Methods). We observed minimal evidence for a difference in *β*_*TCR-mem*_ before and after adjusting for antigen specificity (heterogeneity *p* = 0.45) and minimal heterogeneity in *β*_*TCR-mem*_ across antigen-specific populations (*I*^*2*^ = 29.2%, *H*^*2*^ = 1.41*, Q* = 39.6*, p* = 0.07). TCR-mem associations hold after adjusting for Dextramer staining intensity (STAR Methods) as a proxy for TCR-pMHC affinity, which is thought to contribute to memory T cell development.^49^ These results suggest that TCR-mem sequence features predispose pMHC recognition in general, regardless of the cognate antigen.

### TCR-mem is distinct from antigen binding affinity

While TCR-mem is a property of the TCR sequence alone, binding affinity is a property of a TCR and antigen pair. For a given TCR sequence, binding affinity varies widely with choice of antigen. For example, TCR 1E6^50^ exhibits approximately eight times greater binding affinity to RQFGPDWIVA-HLA-A*02 compared to ALWGPDPAAA-HLA-A*02 (buried surface area: 32,593.7 compared to 3,956.5 Å^2^, PDBePISA). Thus, we did not expect TCR-mem to correspond to binding affinity for any particular antigen.

To assess whether TCR-mem corresponds to TCR-pMHC binding affinity, we (1) conducted tetramer dilution experiments, (2) examined published micropipette adhesion data, and (3) computed buried surface areas in crystal structures. First, we stained each of our four MART-1-specific Jurkat populations with increasing concentrations of MART-1 tetramer (Figure S6C). We approximated each TCR’s affinity to the MART-1 tetramer with EC_5_, the tetramer concentration at which 5% of Jurkat cells stain positively for tetramer. This 5% threshold allowed us to learn dose-response relationships without inducing tetramer aggregation, extrapolating beyond the observed measurements, or generating false positives from negative control tetramers (Figure S6D). EC_5_ measurements for the MART-reactive TCRs did not reveal a clear relationship between TCR-mem and binding affinity (Figure S6E).

Given the potential limitations of tetramers,^51,52^ we next analyzed data from a micropipette adhesion assay that simultaneously measures two-dimensional (2D) TCR affinity and TCR sequence (iTAST).^53^ Compared to tetramers, micropipette adhesion assays are better able to detect low-affinity interactions, and better correspond to gold-standard surface plasmon resonance (SPR) affinity measurements.^51,53^ Moreover, iTAST enables the study of receptors and ligands in their native cell membrane context, without the possible confound of TCR transduction rate. The published iTAST dataset^53^ includes 33 αβ-paired TCRs that bind the hepatitis C virus (HCV) antigen KLVALGINAV complexed with HLA-A*02. We calculated TCR-mem scores for these 33 TCR sequences and again observed no clear relationship between TCR-mem score and binding affinity (*R* = −0.1; Figure S6F).

Third, we analyzed crystal structures of αβTCRs complexed with class I MHC from the Protein Data Bank (PDB). Following quality control, we obtained 138 structures, corresponding to 86 unique epitopes and 17 unique class I HLA alleles. We used PDBePISA to estimate the amount of surface area buried between the TCR and pMHC, a known proxy for binding affinity.^54^ We observed no significant relationship between TCR-mem and binding affinity (*R* = 0.009; Figure S6G). Thus, three lines of evidence (Figures S6E–S6G) indicate minimal correspondence between TCR-mem and binding affinity. TCR-mem appears to capture a TCR characteristic that is distinct from binding affinity yet reliably promotes T cell activation. Investigators have found the relationship between TCR-pMHC binding and T cell activation to be complex.^55,56^

### Thymic selection pressures on the TCR sequence continue in the periphery

Given the consistent association of TCR-mem to memory state across antigenic peptides, we hypothesized that TCR-mem reflects reactivity to the underlying MHC(-like) molecule. Specifically, to promote recognition of many different pMHCs, such reactivity would be focused on elements of MHC that are conserved across MHC genes and alleles. This TCR property, referred to as “generic MHC reactivity,” has been theorized based on thymic development in TCR-transgenic mice.^57^

If TCR-mem reflects generic MHC reactivity, then TCR-mem sequence features should also help to explain which T cells survive thymic positive selection. Only T cells with a sufficient signaling response to pMHC survive thymic positive selection, which is marked by progression from a double-positive (DP) phenotype to a single-positive (SP) phenotype. Thus, if TCR-mem reflects generic MHC reactivity, then SP T cells should have higher TCR-mem compared to DP T cells, 90%^2^ of which never progress to the SP stage.

**Figure 5.**
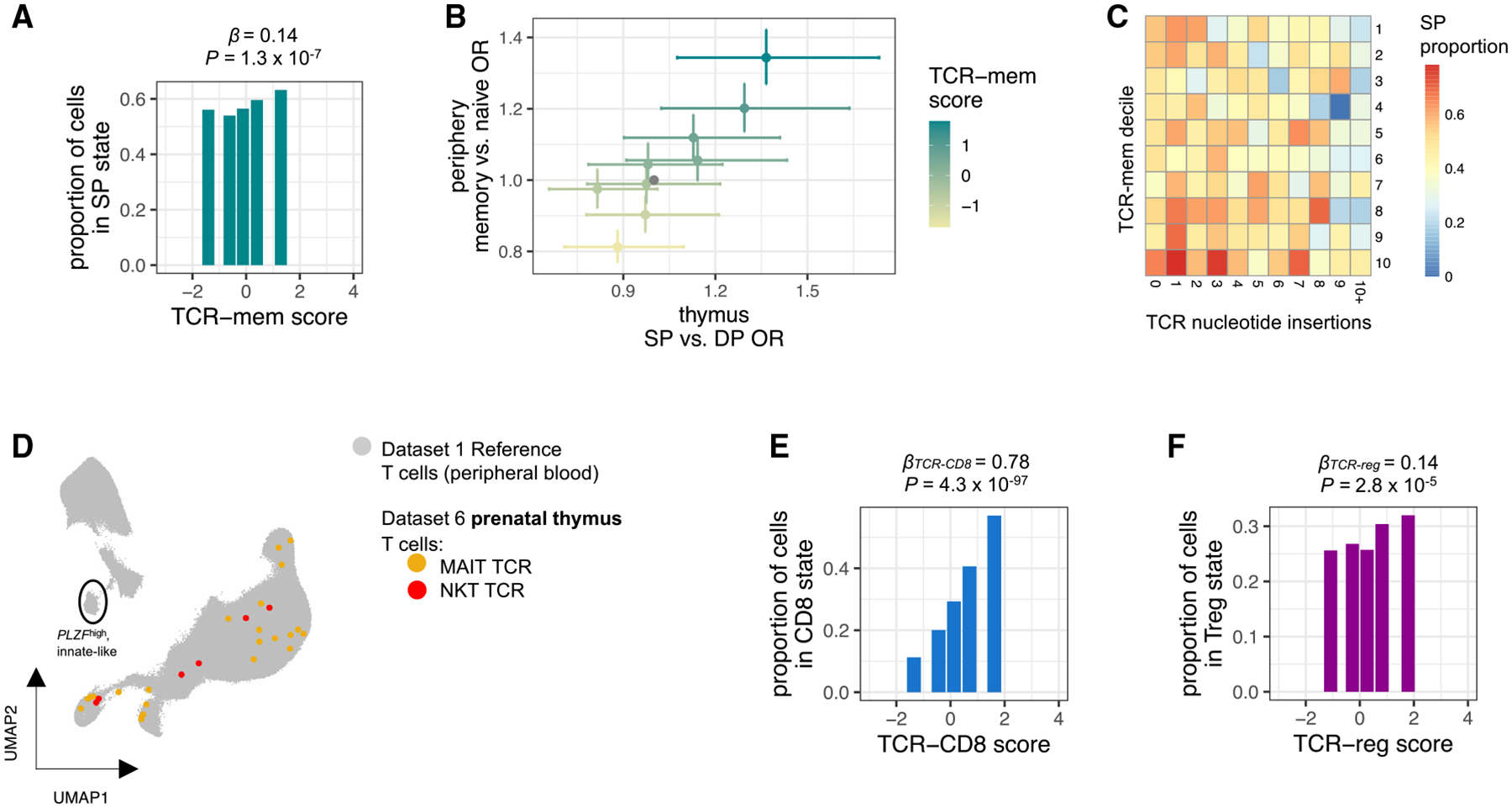
Thymic selection pressures on the TCR sequence continue in the periphery (A) Proportion of SP thymocytes in dataset 6 within each quintile of TCR-mem score. *β* and *p* are computed by mixed-effects logistic regression predicting SP status based on TCR-mem score, *n* = 7,453 thymocytes. (B) Each point represents a TCR-mem decile, plotted according to its association with positive selection in the thymus (dataset 6, x axis) and its association with transitioning from naive to memory in the periphery (dataset 3, y axis). Data are represented as odds ratio ± 95% confidence interval. ORs and 95% CIs are estimated via mixed-effects logistic regression. (C) Heatmap of SP proportion for each combination of TCR-mem score decile and number of TCR nucleotide insertions in dataset 6. (D) Dataset 6 (prenatal thymic) T cells with canonical MAIT cell TCRs (colored yellow, *TRAV1–2* and select *TRAJ* and *TRBV* genes; see STAR Methods) or NKT cell TCRs (colored red, *TRAV10-TRAJ18-TRBV25*) projected into our T cell state reference UMAP constructed from dataset 1 (colored gray). (E) Proportion of SP thymocytes in dataset 6 observed in a CD8 T cell state within each quintile of TCR-CD8 score. *β*_*TCR-CD8*_ and *p* are computed via mixed-effects logistic regression predicting CD8 T cell state based on TCR-CD8 score, *n* = 4,313 SP thymocytes. (F) Proportion of SP thymocytes in dataset 6 observed in a T_reg_ cell state within each quintile of TCR-reg score. *β*_*TCR-reg*_ and *p* are computed via mixed-effects logistic regression predicting T_reg_ cell state based on TCR-reg score, *n* = 4,313 SP thymocytes.

Thus, we compared TCR-mem scores between SP and DP prenatal T cells (14,584 cells, dataset 6, Table 1; STAR Methods). TCR-mem was designed to describe differences between naive and memory TCRs in the periphery. We observed, however, that the same TCR sequence weighting scheme also described differences between DP and SP TCRs (Figure 5A, *β*_*TCR-mem*_ = 0.14, *p* = 1.3 × 10^−7^; Table S4). Strikingly, the TCR-mem difference between SP and DP thymic T cells was statistically indistinguishable from the TCR-mem difference between memory and naive peripheral T cells (heterogeneity *p* = 0.86). Thus, TCR differences between peripheral naive and memory T cells appear to echo TCR filtering by thymic positive selection (Figure 5B). The influence of TCR-mem persists in the absence of foreign and peripheral antigens, suggesting that TCR-mem reflects generic pMHC reactivity. While thymic selection imposes a minimum threshold for pMHC reactivity, TCRs that survive this threshold by a wider margin appear more likely to reach a memory T cell state in the periphery.

An alternative possibility we considered is that higher TCR-mem T cells had developed earlier in life and therefore accrued more opportunities to transition their transcriptional state. This time-related confound would require systematic shifts in V(D)J recombination with human age. However, we observed no relationship between age and TCR-mem (Figure S6H).

Prenatal TCR sequences from dataset 6 allowed us to further extend our age-related line of inquiry. Some prenatal T cells lack *DNTT* expression, precluding non-templated insertion of TCR nucleotides and resulting in systemically shorter TCR sequences.^58^ To identify these age-related TCRs, we applied IGoR^59^ to infer the number of nucleotide insertions in each thymic TCR. We observed that additional nucleotide insertions corresponded to a decrease in the odds of thymic positive selection, but this effect did not account for the effect of TCR-mem (Figure 5C). Controlling for the number of nucleotide insertions, TCR-mem actually demonstrated a stronger effect on the odds of positive selection (conditional *β*_*TCR-mem*_ = 0.22, 95% CI = [0.13–0.30], *p* = 3.8 × 10^−7^, compared to unconditional *β*_*TCR-mem*_ = 0.14, 95% CI = [0.09–0.19], *p* = 1.3 × 10^−7^; Table S4; STAR Methods). Thus, TCR-mem associations are not explained by developmental time points.

In contrast to TCR-mem, TCR-innate was not applicable to thymic data. Thymic T cells expressing canonical TCRs for MAIT cells and NKT cells had not yet reached the innate-like, *PLZF*^high^ transcriptional fate (Figure 5D). This is consistent with previous reports showing that *PLZF*^high^ fate acquisition is dependent on peripheral antigen recognition.^60–62^ TCR-reg and TCR-CD8, however, were applicable to thymic data (Figures 5E and 5F, *β*_*TCR-CD8*_ = 0.78, *p* = 4.3 × 10^−97^; *β*_*TCR-reg*_ = 0.14, *p* = 2.8 × 10^−5^; Table S4; STAR Methods), indicating that these associations between TCR sequence and T cell state are not dependent on peripheral antigen recognition. Evidently, TCR sequence features shape T cell differentiation outcomes in both the thymus and periphery, influencing which T cells are able to generate an effective immune response.

## DISCUSSION

In this study, we define four TCR scoring functions that estimate the TCR’s contribution to four T cell fates. These scoring functions are robust across numerous genetic and clinical contexts. Even among T cells that recognize the same antigen, these TCR scores help to explain variation in T cell states.

Our CCA-based quantitative approach allows us to understand the relative strength of previously observed connection between TCR sequence and T cell state. The most deterministic relationship belongs to MAIT cells. Structural studies have shown that MHC-like molecule MR1 buries small metabolite antigens so that they do not contact TCR.^63,64^ Consequently, MAIT cell TCRs need only recognize MR1, which marks a highly distinct transcriptional population of *PLZF*^high^, innate-like T cells. This tight link from αβ TCR sequence to T cell fate is unusual; were there other relationships of similar magnitude, we believe that our model would have identified them.

We observe elevated CDR3 hydrophobicity in KIR+*HELIOS*+CD8 T cells, consistent with a previous report.^18^ Schattgen et al.^18^ hypothesized that this CD8 population may be “MHC-independent, noncanonical, or self-specific.” Our framework unifies this observation with CD4 investigations^10,16^: KIR^+^CD8^+^ T cells, which may functionally represent human CD8 T_reg_ cells,^36^ appear to resemble CD4 T_reg_ cells in terms of TCR sequence. In general, hydrophobic and aromatic (F, L, I, C, Y, W) junctional CDR3 residues (both α and β) may increase a T cell’s likelihood of recognizing self-antigens, driving *FOXP3* T_reg_ cell fate in the case of CD4 co-receptors and KIR+*HELIOS*+ fate in the case of CD8 co-receptors.

We take particular interest in TCR-mem because it describes TCR sequence features that are generally advantageous for reaching a memory T cell state. Our own tetramer dilution experiments, external micropipette adhesion data, and buried surface area calculations each indicate that TCR-mem is distinct from antigen binding affinity. Rather, our TCR-mem scoring function can distinguish which TCR sequences recognizing a common antigen are more likely to activate. We have shown this with respect to endogenous TCR sequences recognizing a self-antigen (MART-1) and engineered TCR sequences recognizing a viral antigen (pp65). Increased TCR-mem also corresponds to positive selection in the thymus, extending previous observations that T cells with high self-reactivity (CD5^high^) also have higher reactivity to foreign antigens.^65–67^ With TCR-mem, we have identified a common set of TCR sequence features that promote both central and peripheral^68^ selection of the T cell repertoire.

It is now clear that a TCR sequence conveys two types of information: transcriptional fate bias and antigen specificity. Both types of information may be crucial to the immune response in autoimmunity, cancer, and infection. TCR features may play a particularly important role in influencing T cell fate for T cells that recognize autoantigens, which ought to be relatively anergic. The therapeutic design of TCRs may need to consider not only recognition of the antigenic target but also differentiation into an effective T cell state.

### Limitations of the study

There are several limitations to our study. First, our study is restricted to αβ TCR sequences by virtue of the standard custom primer set for V(D)J amplification. The usage of gamma-delta, rather than αβ, TCR genes, has clear effects on the transcriptional state^69^ that merit further study. Second, our approach to defining a standard set of TCR sequence features excluded T cells in which more than one α or one more than one β chain was detected. We expect that dual-α chain T cells follow similar relationships between the TCR sequence and T cell state, but demonstrating this would require separate analysis approaches. Lastly, our TCR scoring functions are consistent across individuals and antigens, but they are insufficient to accurately classify T cell states. After all, the TCR sequence is only one minor influence on the transcriptional state of a given T cell.

## RESOURCE AVAILABILITY

### Lead contact

Further information and requests for resources and reagents should be directed to and will be fulfilled by the lead contact, Soumya Raychaudhuri (soumya@broadinstitute.org).

### Materials availability

Primary materials generated during this study are available upon request through the lead contact.

### Data and code availability

- All sequencing data analyzed in this study were previously deposited in online databases (Table S1).
- Custom analysis code for this manuscript is available at https://github.com/immunogenomics/tcrpheno_analysis. An R package to apply our TCR scoring functions to new data is available at https://github.com/kalaga27/tcrpheno.
- Any additional information required to reanalyze the data reported in this paper is available from the lead contact upon request.

## STAR★METHODS

### EXPERIMENTAL MODEL AND STUDY PARTICIPANT DETAILS

#### Human samples

All human samples analyzed in this study were downloaded from online repositories from prior publications (Table S1), which list demographic characteristics. Sample sizes are listed in Table 1. Human samples were not divided into experimental groups. There was minimal influence of sex on the results of the study (Table S4).

#### Cell lines

TCRβ-null Jurkat (J.RT3-T3.5, male) and HEK-293T (CRL-3216, female) cell lines were obtained from the American Type Culture Collection (ATCC). HEK-293T cells were engineered to express HLA-A2 and authenticated by flow cytometry as described previously.^70^ TCRβ-null Jurkat cells were authenticated by flow cytometry, verifying that they do not express TCR at the cell surface. TCRβ-null Jurkat cells were cultured in RPMI (Gibco, A10491-01) with 10% FBS (HyClone) and 1% penicillin-streptomycin (Invitrogen, 15140-122). HEK-293T cells were cultured in DMEM (Gibco, 11995065) with 10% FBS (HyClone) and 1% penicillin-streptomycin (Invitrogen, 15140- 122). Cells were regularly tested for mycoplasma; results were always negative.

### METHOD DETAILS

#### Generation of antigen-specific Jurkat cell lines

TCRβ-null Jurkat cells were spinfected with lentivirus at varying concentrations to achieve a multiplicity of infection (MOI) < 1 and introduced with TCRs of interest. Each TCR was encoded by a single construct containing the mouse TCR constant region. A total of 1 × 106 cells were spun with 8 μg mL–1 polybrene (Millipore, TR-1003-G) and lentivirus for 30 min at 800g in 12-well plates. Cells were incubated at 37°C and the virus was washed off after 24 h. After spinfection for 48 h, surface expression of TCR constructs was confirmed by flow cytometry.

#### Tetramer generation

The HLA-A*02:01 MART-1 and pp65 peptides were synthesized by Genscript and loaded onto APC QuickSwitch Quant HLA-A*02:01 tetramers (MBL International). Negative control tetramers were prepared with Tax peptide (LLFGYPVYV) and loaded onto PE QuickSwitch Quant HLA-A*0201 tetramers (MBL International).

#### Endogenous antigen expression cocultures

Following 8–16-h incubation at 37°C, cells were washed twice with PBS supplemented with 2 mM EDTA (PBE) and 0.5% BSA. For endogenous antigen expression, 56-mer peptide fragments were reverse-translated and synthesized as gBlocks (IDT) with 5′ and 3′ BP recombination sites and cloned into pDONR221. Peptide antigens were subsequently cloned into pHAGE-CMV-Nflag-HA-DEST-IRES-Puro destination vectors using LR clonase (ThermoFisher Scientific Gateway Clonase).

pp65 56-mer: RLKAESTVAPEEDTDEDSDNEIHNPAVFTWPPWQAGILARNLVPMVATVQSGARA*

MART-1 56-mer: MPREDAHFIYGYPKKGHGHSYTTAEEAAGIGILTVILGVLLLIGCWYCRRRNGYRA

#### Flow cytometry

All antibodies were purchased from BioLegend and were used at 1 μL per million cells. Cells were stained with antibodies for at least 30 min in PBE and then washed in PBE twice. Samples were acquired through the CytoFLEX (Beckman Coulter) flow cytometer and data were analyzed through FlowJo (version 5.0.0) software. When screening for Jurkat activation following coculture with 293T cells, Jurkat cells were sorted from 293T cells based on CD40 expression (APC anti-mouse CD40 clone 3/23), and then stained for CD69 expression (PE anti-human CD69, clone FN50). For tetramer dilution experiments, CD3^high^ Jurkat cells (BV421 anti-human CD3, 300434) were isolated, and then stained for tetramer positive-control (APC QuickSwitch, MBL International) and tetramer negative-control (PE QuickSwitch, MBL International).

### QUANTIFICATION AND STATISTICAL ANALYSIS

The details of each statistical test can be found in the corresponding figure legend.

#### Sequencing read alignment

Sequencing data were deposited and downloaded as aligned sequencing reads (raw UMI counts for genes, ADTs, or TCR contigs) for Datasets 1–4, and Dataset 6. For TCRs in Dataset 4, we downloaded .fastqs from the European Nucleotide Archive (ENA). We used cellranger vdj version 5.0.1 to align reads to reference GRCh38 5.0.0.

#### Quality control and normalization

For all single-cell datasets, we removed cells with UMIs for less than 500 genes and cells with greater than 10% of UMIs derived from mitochondrial genes. We then normalized UMIs for each gene in each cell to log(UMI counts per 10,000) and normalized UMIs for each surface protein in each cell via the centered-log-ratio (CLR) transformation.

##### Unimodal dimensionality reduction

To identify clusters A1–A9, we applied a unimodal dimensionality reduction pipeline to Dataset 1, using mRNA information alone. After normalizing gene expression, we used the variance-stabilizing transformation (VST) to select the 200 most variable genes in each sample, excluding TCR genes. The union of variable genes across samples totaled 6358 genes. To conduct principal component analysis (PCA), we used R package “irlba” (v2.3.3) on normalized expression of these 6358 genes, each scaled to mean 0 variance 1. We then used the R package Harmony^71^ (v1.0) to remove batch effects from the per-cell principal component scores. We applied Harmony to three batch variables: ‘scRNASeq_sample_ID’, ‘Institute’, and ‘Pool_ID.’ We used θ values 1, 0.5 and 0.5 for these batch variables, respectively. We used UMAP to visualize the resultant cell embedding in two dimensions, computed through the R package Symphony^72^ (v1.0). We built a shared-nearest-neighbor (SNN) graph of cells based on the first 20 batch-corrected gene expression PCs, using R package “singlecellmethods” (v0.1.0, parameters: prune_snn = 1/25, nn_k = 10, nn_eps = 0.5). To identify transcriptional clusters, we applied Louvain clustering to the SNN graph at multiple resolutions, through the *RunModularityClustering* function from the R package Seurat (v3.2.2). Clusters A1–A9 were identified by clustering resolution 0.5.

##### Multimodal dimensionality reduction

To identify clusters B1–B9, we applied a multimodal dimensionality reduction pipeline^27^ to Dataset 1, incorporating both mRNA and surface protein information. We used the VST to select the 100 most variable genes in each sample, excluding TCR genes. The union of variable genes across samples totaled 4423 genes. We applied Canonical Correlation Analysis (CCA) to paired mRNA and protein measurements, using the expression of these 4423 genes as input matrix 1 and the expression of 10 surface proteins as input matrix 2 (R package “CCA” v1.2.1). To emphasize traditional T cell populations, we selected TotalSeq antibodies for 10 surface proteins critical to distinguish between CD4, CD8, central memory (CM) and effector memory (EM) T cells: CD4, CD8, CD45RO, CD45A, CD197_CCR7, CD62L, CD27, CD11a, CX3CR1, and KLRG1. Input gene expression was log10CPK-normalized and scaled to mean 0 variance 1; input protein expression was CLR-normalized and scaled to mean 0 variance 1. As in our unimodal dimensionality reduction pipeline, we then removed batch effects from the per-cell mRNA-based canonical variate (CV) scores, constructed a UMAP and SNN graph based on the 10 batch-corrected CVs, and identified cell clusters. Clusters B1–B9 were identified through clustering at resolution 4.0, and collapsing clusters with similar marker expression (Figures S2Q and S2R).

##### Standardizing T cell states across datasets

To annotate T cell states in a consistent manner across datasets, we conducted reference mapping with Symphony^72^ (v1.0). Our unimodal T cell state reference (Figure S1) and multimodal T cell state reference (Figure S2) were constructed from Dataset 1 (T cells in peripheral blood). To annotate T cell states in Datasets 2–5, we projected cells into these references. Symphony’s reference mapping includes correction for batch variables, which we specified for each dataset. For all datasets, we corrected for the individual’s donor ID. For Dataset 2, we additionally corrected for batch variables “Sample.type” and “PMID.” For Dataset 3, we additionally corrected for batch variable “Site.” For Dataset 4, we additionally corrected for batch variable “Chemistry,” and conducted Symphony mapping separately for each tissue.

For each projected cell, we used the R package “class” (v7.3.17) to identify the five nearest neighbor cells from Dataset 1. We transferred the majority cell state label among these five to the projected cell.

We did not transfer cell state annotations for Dataset 6, because prenatal thymic tissue includes progenitor T cell states not observed in peripheral blood. Instead, we used T cell state annotations provided by the authors.^24^

#### Quality control for TCR sequence data

TCR sequence data deposited for Dataset 2 included only cells with exactly one TCRα chain and exactly one TCRβ chain, with Vα, Jα, Vβ, and Jβ gene names resolved. In keeping with this quality control by Ren et al.,^20^ we filtered all other scRNAseq-TCR datasets to include only TCRs with exactly one TCRα chain, exactly one TCRβ chain, and Vα, Jα, Vβ, and Jβ gene names resolved. For analyses purely focused on cell state, such as dimensionality reduction and clustering, we did not apply TCR-based filtering.

#### Defining TCR clonotypes

To define the TCR clonotype for each cell, we concatenated the IMGT Vα, Jα, Vβ, and Jβ gene names with the CDR3ɑ amino acid sequence and CDR3β amino acid sequence. To be considered a “TCR-twin,” TCRs from two different individuals were required to match exactly for each of these TCR components. To be considered part of the same expanded clone, TCRs had to match exactly for each of these TCR components, and be sampled from the same individual. We recognize that it is optimal to identify expanded clones by nucleotide rather than amino acid sequence, because different ancestral V(D)J nucleotide recombinations can converge to the same amino acid sequence. However, only amino acid sequences were made publicly available for the TCRα chain for Dataset 1. In practice, because amino acid convergence is rare, TCR clones are often identified via amino acid sequence.^19^

##### TCR twin analysis

To identify “TCR twins” in Dataset 1, we counted the number of individuals observed for each TCR clonotype. The vast majority of TCR clonotypes (234131/234265, 99.9%) were observed in only one individual from Dataset 1, and would be considered “private TCRs.” 134 TCR clonotypes were observed in more than one individual (“public TCRs”), and we analyzed the 115 TCR clonotypes that were observed in exactly two individuals.

We next assigned a transcriptional state to each TCR twin member. 115 TCR clonotypes, each observed in two individuals, implies 230 twin members. For 163/230 twin members (70%), we observed no evidence of clonal expansion (there was only one cell sampled from the individual with the TCR clonotype of interest), and we used the cluster identity of its single cell. For 37 twin members (16%), we observed clonal expansion within a single transcriptional cluster (resolution 0.5, Figure 1A), and we used this cluster to annotate the TCR twin member. For 30 twin members (13%), we observed clonal expansion across multiple clusters, and we used the transcriptional cluster that contained the greatest number of constituent cells.

We then counted how many of the 115 TCR twins were assigned the same transcriptional cluster in both individuals. To assess statistical significance, we conducted a binomial test with *N* = 115 trials and *P*_*null*_ = the probability of concordant transcriptional clusters by chance. To calculate *P*_*null*_, we summed the probabilities of randomly drawing two observations with the same cluster assignment for each of the nine transcriptional clusters (A1–A9, Figure 1A). For transcriptional cluster *j* including *n*_*j*_ of the 230 T cells with twinned TCRs, this probability can be computed as:


(Equation 1) $$P_{j}=\frac{\left(\begin{array}{c} n_{j}\\ 2 \end{array}\right)}{\left(\begin{array}{c} 230\\ 2 \end{array}\right)}$$

*P*_*null*_ can then be found by summation:

(Equation 2) $$P_{\text{null}}=\sum_{j=1}^{9}P_{j}$$

##### TCR featurization

Cellranger vdj output provides amino acid sequence for CDR3 regions, but IMGT gene names only for other regions of the TCR. To translate IMGT gene names into CDR1 and CDR2 amino acids, we downloaded amino acid sequences for each gene from https://www.imgt.org.

We removed all TCR sequences for which the V gene is unresolved. To focus on functional TCRs, we removed V genes listed as pseudogenes in IMGT. We used TCR position numbers from IMGT to describe the location of each TCR amino acid. To maintain a tractable number of TCR positions, we consider only TCR sequences with CDR3a length ranging from 10 to 17 amino acids and CDR3b length ranging from 11 to 18 amino acids.

To convert each TCR amino acid into a vector of quantitative features, we translated each TCR amino acid into its five Atchley factors.^30^ This process results in 290 TCR features, extracted from the 58 TCR amino acid positions that comprise CDR1a, CDR2a, CDR3α, CDR1b, CDR2b, and CDR3b. We also computed the percentage of each CDR loop occupied by 19 of the 20 amino acids, excluding Glycine as a reference. We also included six TCR features corresponding to the length (in amino acids) of each of the CDR loops. Finally, for each pair of adjacent residues within a TCR chain, we multiplied each possible combination of the five Atchley factors, resulting in 25 interactive TCR features per pair of adjacent residues. The resultant list of 1250 TCR sequence features is listed in Table S2. For all analyses, we scaled each TCR feature such that it would have mean 0 and variance 1 in the training dataset. Because the number of amino acids in a TCR sequence varies from cell to cell, shorter TCR sequences contain gaps at some IMGT positions. We fill these entries with the value 0, following the scaling transformation.

##### Developing TCR scoring functions

For our purposes, a TCR scoring function takes an amino acid TCR sequence as input and returns a numeric value proportional to the odds of that TCR being observed in the T cell state of interest. This transformation is accomplished by a set of TCR sequence feature weights, which can be learned from our training observations (70% of clones in Dataset 1 and Dataset 2). After training, the TCR sequence feature weights were fixed, and the resultant TCR scoring function can be applied to external data (e.g., Datasets 3–6).

We considered three methods to construct TCR scoring functions:

Method 1: Regularized Canonical Correlation Analysis (rCCA)

By identifying axes of covariation between TCR sequence features and T cell state features across cells, rCCA produces a series of correlated TCR and T cell state scoring functions. rCCA does not require the analyst to pre-specify T cell states of interest. Instead, it identifies continuous T cell states, which may not exactly align with preexisting T cell state definitions. We applied rCCA via R package “mixOmics” (v6.19.1), tuning ridge penalties via 5-fold cross-validation. For ease of interpretation, we reversed the sign of CV1 scores and CV3 scores.

Method 2: Logistic Regression with Ridge Penalty

Results from rCCA clearly pointed to four recognizable T cell states. rCCA-based TCR scoring functions are optimized to predict the continuous-valued T cell states identified by rCCA, rather than recognizable and reproducible T cell state distinctions. Thus, to enhance biological interpretability, we translated the continuous T cell state values identified by rCCA into binary contrasts: 1) whether the cell belonged to transcriptional cluster A9 (innate-like T), 2) whether the cell belonged to transcriptional clusters B1–B3 (CD8T), 3) whether the cell belonged to the union of transcriptional cluster A5 or transcriptional cluster C15 (CD4^+^ T_reg_ and CD8^+^ T_reg_, respectively, Figure S3K), and 4) whether the cell did not belong to transcriptional clusters B3 or B9 (memory T). We fit a logistic regression to each of these four binary T cell state contrasts:

(Equation 3) $$\text{logit}\left(TCS_{i}\right)=\left(\sum_{j=1}^{1250}\beta_{j}\text{TCR}\;\text{featur}e_{j,i}\right)+\beta_{0}+\varepsilon_{i}$$

Each observation *i* represents one T cell. Consistent with our input to rCCA, we used one cell selected at random to represent each expanded clone. *TCS*_*i*_ equals 1 if cell *i* is observed in the T cell state of interest; 0 otherwise.

We used the same 1250 TCR sequence features as predictors, and the same set of training observations as in Method 1. To handle collinearity between these TCR sequence features, we implemented ridge regularization via R package “glmnet” (v4.0.2), using 5-fold cross-validation within the training set to select the optimal penalty weight. We removed innate-like cells (transcriptional cluster A9) from regressions focused on CD8T, T_reg_, or memory T cell fate.

Method 3: Convolutional Neural Network (CNN) Binary Classifier.

Methods 1 and 2 only consider linear effects of TCR sequence features and interactive effects limited to adjacent TCR residues. To assess whether nonlinear and/or motif-based TCR sequence feature effects would improve T cell state predictions, we implemented a Convolutional Neural Network (CNN) binary classifier for each of the four T cell state contrasts described above. In the CNN framework, each TCR sequence is represented by a 5 × 58 matrix, corresponding to 5 Atchley factors at each of the 58 residues comprising CDR1a, CDR2a, CDR3a, CDR1b, CDR2b, and CDR3b. After applying convolution and average pooling to this matrix, we concatenate the resultant hidden layer to the TCR score learned through logistic regression. This ensures that the CNN does not have to re-learn linear effects, and can instead focus on potential nonlinear and motif-based effects. We implemented these CNN binary classifiers via python package “torch” (v1.4.0), using the same training and testing split (within Dataset 1 and Dataset 2) as in Method 1 and 2. We used the Adam optimizer and the binary cross-entropy with logits loss function (“BCEWithLogitsLoss” from python package torch), and iterated over a grid of hyperparameters corresponding to batch size, learning rate, and size of hidden layers. For each of these hyperparameter combinations for each of the four cell state contrasts (3 × 2 × 3 × 4 = 72 models), we trained a CNN on Datasets 1 and 2, and applied the resultant classifier to Dataset 3. We observed minimal impact of hyperparameters on AUC, and therefore selected the least complex model (10 nodes in hidden layer, learning rate 0.0003, and batch size of 256). We used early stopping^73^ to mitigate overfitting, training only until classification performance stopped improving in the held-out testing data (Figures S5A–S5D).

*Benchmarking:* To benchmark these different TCR scoring functions methods, we applied them to a dataset external to training (Dataset 3), and scaled each type of TCR score to have mean 0 variance 1 prior to association testing via Equation 4.

##### Applying TCR scoring functions to data outside of the training set

To apply the TCR scoring functions to data outside of the training set, we extracted the same 1250 TCR sequence features, multiplied each TCR feature value by its respective weight learned in from Dataset 1 and 2, and took the sum of these 1250 products. For each TCR feature value, we subtracted its mean value in Datasets 1–2 and divided the result by its standard deviation in Datasets 1–2. Similarly, for each summation over TCR feature value-weight products, we subtracted the Dataset 1–2 mean and divided the result by the Dataset 1–2 standard deviation. Thus, one unit of TCR-mem in any dataset corresponds to one standard deviation of TCR-mem in Datasets 1–2.

##### TCR sequence feature dependence analysis

Given the correlation structure of TCR sequence features, we iteratively masked out correlated blocks of features using the Partition Explainer of the python package “shap”. To estimate the relative contribution of each CDR loop, we took the absolute value of Shapley scores and summed over examples in the positive class for each TCR scoring function.

##### Association testing between TCR and T cell state

To test for an association between a TCR score and T cell state (TCS) contrast of interest, we used mixed-effects logistic regression. Using R package “lme4” (v1.1.23), we fit the following regression:

(Equation 4) $$\text{logit}\left(TCS_{i}\right)=\beta_{\text{TCR}\;\text{score}}\cdot \text{TCR}\;\text{scor}e_{i}+\beta_{0}+\beta_{0j}+\varepsilon_{i}$$

Each observation *i* represents one T cell from individual *j*. Consistent with our input to rCCA, we used one cell selected at random to represent each expanded clone. *TCS*_*i*_ equals 1 if cell *i* is observed in the T cell state of interest; 0 otherwise. *β*_*0*_ is the global intercept; *β*_*0j*_ is a modification to the global intercept fit for each individual *j*. *TCRscore*_*i*_ represents TCR score values, which are standardized to have mean 0 and variance 1 in the training observations from Dataset 1 and Dataset 2.

To estimate *β*_*TCR-innate*_*, β*_*TCR-CD8*_*, β*_*TCR-reg*_*, and β*_*TCR-mem*_ separately for each individual from Dataset 1 and Dataset 2 (Figures S4I–S4L), we followed the same procedure with the removal of the *β*_*0j*_ term. Removing *β*_*0j*_ reduces the mixed-effects logistic regression to a logistic regression, requiring R package “stats” (v3.6.3) rather than “lme4” for parameter estimation. We included only individuals with at least 100 cells including at least 10 cells that matched the cell state of interest and at least 10 cells that did not match the cell state of interest in the regression. Then, to estimate the distribution of *β*_*TCR-innate*_ across individuals, we used to these per-individual estimates as input to R function “rma” (from R package “metafor”, v4.0.0), using the maximum likelihood estimator of heterogeneity (method = “ML”).

##### Analysis of nonproductive TCRs

To test whether our TCR scoring functions explained T cell state only when applied to the surface-expressed TCR, we analyzed nonproductive TCRs from blood and secondary lymphoid tissue (Dataset 4). We collected all contigs annotated as high confidence, full length, and nonproductive by cellranger vdj (v5.0.1). Many nonproductive TCRs are short in length due to a stop codon; accordingly, we lifted our minimum CDR3 amino acid requirement. Because nonproductive TCRs are lowly expressed, it is uncommon for both a nonproductive alpha chain and a nonproductive beta chain to be detected in the same cell. Thus, to attain statistical power, we did not require cells to have both a nonproductive alpha and a nonproductive beta chain. Instead, for each of our TCR scoring functions, we created a version that used only TCR alpha chain information and a version that used only TCR beta chain information (4 × 2 = 8 single-chain TCR scoring functions). As before, we used productive TCRs from Dataset 1 and Dataset 2 to train TCR feature weights. We then applied these single-chain TCR scoring functions to nonproductive TCRs from Dataset 4. We removed clonal expansion from Dataset 4 as before, using productive TCR sequences to define T cell clones and selecting one cell at random to represent each expanded clone. We then tested for associations to T cell state as outlined by Equation 4. To confirm that lack of association for nonproductive TCRs was not due to statistical power or dataset, we down-sampled productive TCRs from Dataset 4 to match the sample size of nonproductive TCRs and repeated association testing (Table S4).

##### TCR-mem within antigen-specific populations

To stringently remove background Dextramer counts, we fit a negative binomial regression for each of the 44 Dextramers, estimating contributions from technical factors:

(Equation 5) $$\text{log}\left(dUMI_{i}\right)=\left(\sum_{k=1}^{6}\beta_{k}\cdot ncUMI_{k,i}\right)+\beta_{\text{TCRexp}}{\text{TCRexp}}_{i}+\beta_{CD3\;\text{exp}}CD3\;{\text{exp}}_{i}+\beta_{CD8\;\text{exp}}CD8\;{\text{exp}}_{i}+\left(\sum_{j=2}^{4}\beta_{j}\text{dono}r_{j,i}\right)+\beta_{0}+\varepsilon_{i}$$

*dUMI*_*i*_ denotes the count of UMIs for cell *i* for the Dextramer of interest. *ncUMI*_*k*;*i*_ denotes the count of UMIs for negative control Dextramer *k* collected for cell *i*. We include *β*_*TCRexp*_*TCRexp*_*i*_ and *β*_*CD*3_ *expCD*3 *exp*_*i*_ to correct for TCR expression level, as more TCRs expressed at the cell surface should render more opportunities to bind Dextramer. *TCRexp*_*i*_ equals the log(CP10K + 1) normalized expression for CDR3 UMIs (alpha and beta summed) for cell i; *CD*3*exp*_*i*_ equals the CLR-normalized expression of the CD3 protein for cell i. We include *CD*8 *exp*_*i*_ (CLR-normalized expression of the CD8 protein for cell i) to adjust for the possibility that greater co-receptor expression renders more opportunities to bind Dextramer. We used R package “MASS” (v7.3.54) to fit the negative binomial regressions.

To normalize Dextramer staining values, we replaced each cell’s raw Dextramer UMI count with an expression of its negative binomial regression residual:

(Equation 6) $$\text{Dnor}m_{i}=\text{log}\left(\frac{\text{observed}\;{\text{UMI}}_{i}}{\text{expected}\;{\text{UMI}}_{i}}+1\right)$$

*Dnorm*_*i*_ equals the normalized Dextramer staining value for cell *i*. *expected UMI*_*i*_ equals the negative binomial prediction based on technical factors for cell *i*. For most Dextramers, we observed a bimodal distribution of normalized Dextramer staining values, distinguishing a background population from an antigen-binding population with Dextramer UMI counts than could be attributed to technical factors. T cells in this putative antigen-specific population were less likely to have other Dextramers with non-zero UMI counts (Figures S7A and S7B). By careful visual inspection, we set a normalized Dextramer staining threshold for each antigen-specific population to distinguish binders from non-binders (Figures S7A–S7B).

If T cells assigned to the same antigen specificity based on Dextramer UMI counts really do share antigen specifiity, they should have similar TCR sequences. Thus, we turned to TCR sequences to validate our inferences about antigen specificity. We observed significant TCR sequence conservation within our inferred antigen-specific populations (Figures S7C and S7D).

Within each group of T cells assigned the same antigen specificity, we fit the following logistic regression (R package stats v3.6.3):

(Equation 7) $$\text{logit}\left(\text{memor}y_{i}\right)=\beta_{\text{TCRmem}}\;\text{TCRme}m_{i}+\beta_{\text{Dnorm}}D_{\text{nor}m_{i}}+\left(\sum_{j=2}^{4}\beta_{j}\text{dono}r_{j,i}\right)+\beta_{0}+\varepsilon_{i}$$

memory_*i*_ takes the value 1 if T cell *i* is observed in a memory state (clusters B1, B2, B4) and value 0 if T cell *i* is observed in a naive state (cluster B3). As in previous analyses, we chose one cell at random to represent each expanded clone. To adjust for affinity between the TCR and Dextramer in question, we include *Dnorm*_*i*_ as a covariate, equal to the extent of Dextramer staining following our custom normalization for cell i (see Equation 6). For antigen-specific populations that span more than one of the four donors in Dataset 5, we include donor covariate terms to adjust for donor-specific effects. We required that the antigen-specific population have at least 10 distinct TCR clones. Altogether, this process yielded 29 estimates of *β*_*TCR – mem*_ for 29 groups of T cells, each specific to a different Dextramer.

We next wanted to understand the typical value of *β*_*TCR – mem*_ in any antigen-specific T cell population. With the 29 antigen-specific *β*_*TCR – mem*_ estimates and their standard errors, we conducted a random-effects meta-analysis (R package “metafor”, v4.0.0). We used the maximum likelihood estimator of heterogeneity (method = “ML”).

## Supplementary Material

Supplementary Information (Including Supplementary Figures)

Table S2

Table S3

Table S4

## Figures and Tables

**Table 1.** Datasets analyzed in this study

Name	Reference	Sample size and attributes	No. of T cells after quality control	Purpose
Dataset 1	COMBAT^19^	77 individuals with COVID-19, 23 with sepsis, 12 with influenza, 10 healthy controls	282,639	twin TCR analysis, developing TCR scoring functions
Dataset 2	Ren et al.^20^	117 individuals with COVID-19, 17 healthy controls	211,780	developing TCR scoring functions
Dataset 3	Stephenson et al.^21^	89 individuals with COVID-19, 26 healthy controls	144,175	validating TCR scoring functions
Dataset 4	Dominguez Conde et al.^22^	8 organ donors	30,702	validating TCR scoring functions
Dataset 5	Boutet et al.^23^	4 heathy individuals	136,546	assessing TCR scoring functions within antigen-specific populations
Dataset 6	Suo et al.^24^	6 prenatal samples	13,930	assessing TCR scoring functions in the thymus
HLA-genotyped dataset	Su et al.^25^	129 individuals with COVID-19	141,759	twin TCR analysis, accounting for HLA genotypes

List of published single-cell datasets used in this study, their attributes, and their use.

**Table 2.** TCR sequences used in transduction experiments

ID	Cognate antigen	Vα	Jɑ	CDR3α	Vβ	Jβ	CDR3β	TCR-mem score
MART-a	MART-1	TRAV12–2	TRAJ45	CGVSGGGADGLTF	TRBV6–2	TRBJ2–7	CASTDSPGLAGGYEQYF	−2.25
MART-b	MART-1	TRAV12–2	TRAJ31	CAGNNARLMF	TRBV28	TRBJ1–5	CASRGTGLGNQPQHF	−0.92
MART-c	MART-1	TRAV12–2	TRAJ17	CAVKNAGNKLTF	TRBV7–2	TRBJ2–7	CASSLNDFYEQYF	−0.02
MART-d	MART-1	TRAV12–2	TRAJ11	CASSWGGYSTLTF	TRBV20–1	TRBJ2–7	CSARVETSGIHEQYF	1.35
NLV-a	pp65	TRAV35	TRAJ50	CAGP**MITSQ**DKVIF	TRBV12–4	TRBJ1–2	CASSSANYGYTF	−0.84
NLV-b	pp65	TRAV35	TRAJ50	CAGP**MLTSQ**DKVIF	TRBV12–4	TRBJ1–2	CASSSANYGYTF	−0.59
NLV-c	pp65	TRAV35	TRAJ50	CAGP**NPTTY**DKVIF	TRBV12–4	TRBJ1–2	CASSSANYGYTF	−0.11
NLV-d	pp65	TRAV35	TRAJ50	CAGP**MKTSY**DKVIF	TRBV12–4	TRBJ1–2	CASSSANYGYTF	0.03

List of TCR sequences used in transduction experiments.

**Table:** KEY RESOURCES TABLE

REAGENT or RESOURCE	SOURCE	IDENTIFIER
Antibodies		
APC anti-mouse CD40, clone 3/23	BioLegend	Cat# 124637; RRID:AB_2860658
PE anti-human CD69, clone FN50	BioLegend	Cat# 310906; RRID:AB_314841
BV241 anti-human CD3	BioLegend	Cat# 300434; RRID:AB_10962690
Chemicals, peptides, and recombinant proteins		
MART-1 peptide	Genscript	N/A
pp65 peptide	Genscript	N/A
Tax peptide	Genscript	N/A
APC QuickSwitch Quant HLA-A*02:01	MBL International	Cat# TB-7308-K2
PE QuickSwitch Quant HLA-A*02:01	MBL International	Cat# TB-7308-K1
Experimental models: Cell lines		
HEK-293T	American Type Culture Collection	CRL-3216
TCRβ-null Jurkat	American Type Culture Collection	J.RT3-T3.5
Software and algorithms		
R package tcrpheno	https://github.com/kalaga27/tcrpheno	Zenodo: https://doi.org/10.5281/zenodo.14205113
Manuscript analyses	https://github.com/immunogenomics/tcrpheno_analysis	Zenodo: https://doi.org/10.5281/zenodo.14213211

## Highlights

- Machine learning with scRNA-seq elucidates links between TCR sequence and T cell function
- Four TCR scoring functions quantify T cell fate predispositions encoded in the TCR
- A common set of amino acid features enhances activation of TCRs for different antigens
