# Supplementary material for: The T cell receptor sequence influences the likelihood of T cell memory formation
Source: Cell Rep. Author manuscript; Available in PMC 2025 Feb 1. (PMC11785489; doi:10.1016/j.celrep.2024.115098)
Supplement: Supplementary Information (Including Supplementary Figures) [file NIHMS2046529-supplement-Supplementary_Information__Including_Supplementary_Figures_.pdf]

Cell Reports, Volume *44*

Supplemental information

The T cell receptor sequence influences the likelihood of T cell memory formation

Kaitlyn A. Lagattuta, Ayano C. Kohlgruber, Nouran S. Abdelfattah, Aparna Nathan, Laurie Rumker, Michael E. Birnbaum, Stephen J. Elledge, and Soumya Raychaudhuri

Table S1. Download information for datasets used in this study, related to Table 1

Name	Reference	URL	Data type	File	Date of download
Dataset 1	COMBAT	https://doi.org/10.5281/zenodo.6120249	Sample metadata	CBD-KEY-CLINVAR.tar.gz	6/23/22
			RNA counts	COMBAT-CITESeq-DATA.h5ad	6/23/22
			ADT counts	COMBAT-CITESeq-DATA.h5ad	6/23/22
			TCR	CBD-KEY-CITESEQ-VDJ-T.tar.gz	6/23/22
Dataset 2	Ren et al.	https://www.ncbi.nlm.nih.gov/geo/query/acc.cgi?acc=GSE158055	Sample metadata	GSE158055_sample_metadata.xlsx	11/21/21
			RNA counts	GSE158055_covid19_counts.mtx.gz	11/21/21
			Cell barcodes	GSE158055_covid19_barcodes.tsv.gz	11/21/21
			Gene names	GSE158055_covid19_features.tsv.gz	11/21/21
			TCR	GSE158055_covid19_BCR_TCR.tar.gz	11/21/21
			Cell annotations	GSE158055_cell_annotation.csv.gz	11/21/21
Dataset 3	Stephenson et al.	https://www.ebi.ac.uk/biostudies/arrayexpress/studies/E-MTAB-10026	Sample metadata	covid_portal_210320_with_raw.h5ad	3/22/21
			RNA counts	covid_portal_210320_with_raw.h5ad	3/22/21
			ADT counts	covid_portal_210320_with_raw.h5ad	3/22/21
			TCR	TCR_merged-Updated	3/28/23
Dataset 4	Domínguez Conde et al.	tissueimmunecellatlas.org	Sample metadata	conde_t-cells.h5ad	8/2/22
			RNA counts	conde_t-cells.h5ad	8/2/22
		European Nucleotide Archive: ftp.sra.ebi.ac.uk/vol1/run/ERR924	TCR	See Supplementary Table 13	8/2/22
Dataset 5	Boutet et al.	https://www.10xgenomics.com/resources/datasets/cd-8-plus-t-cells-of-healthy-donor-1-1-standard-3-0-2	Sample metadata	vdj_v1_hs_aggregated_donor[X]/vdj_v1_hs_aggregated_donor[X]_binarized_matrix.csv *for [X] 1-4*	8/30/22

			Dextramer counts	vdj_v1_hs_aggregated_donor[X]/vdj_v1_hs_aggregated_donor[X]_binarized_matrix.csv<br>*for [X] 1-4*	8/30/22
			ADT counts	vdj_v1_hs_aggregated_donor[X]/vdj_v1_hs_aggregated_donor[X]_filtered_feature_bc_matrix.tar.gz<br>*for [X] 1-4*	8/30/22
			RNA counts	vdj_v1_hs_aggregated_donor[X]/vdj_v1_hs_aggregated_donor[X]_filtered_feature_bc_matrix.tar.gz<br>*for [X] 1-4*	8/30/22
			TCR	vdj_v1_hs_aggregated_donor[X]/vdj_v1_hs_aggregated_donor[X]_all_contig_annotations.csv<br>*for [X] 1-4*	8/30/22
Dataset 6	Suo et al.	https://developmental.cellatlas.io/fetal-immune	Sample metadata	PAN.A01.v01.raw_count.20210429.NKT.embedding.h5ad	12/10/22
			RNA counts	PAN.A01.v01.raw_count.20210429.NKT.embedding.h5ad	12/10/22
			TCR	PAN.A01.v01.raw_count.20210429.NKT.embedding.abTCR.h5ad	12/10/22
HLA-genotyped dataset	Su et al.	https://www.ebi.ac.uk/biostudies/arrayexpress/studies/E-MTAB-9357	HLA genotype, GEX, and TCR	individual files per sample	11/10/23

Table S5.Cell state frequencies in Dataset 3, related to Figure 3

Dataset	TCR score	cell state of interest	frequency of cell state of interest in top percentile of TCR score	frequency of cell state of interest in bottom percentile of TCR score
Dataset 3	TCR-innate	innate-like (PLZF-high)	0.911	0.00659
Dataset 3	TCR-CD8	CD8T	0.716	0.0271
Dataset 3	TCR-reg	Treg	0.00869	0.0541
Dataset 3	TCR-mem	memory	0.59	0.42

Table S6. Comparison between TCR scoring function methods, related to Figure S5

data	method	TCR score	cell state contrast	value	term	estimate	95% CI
Dataset 3	regularized logistic regression	TCR-innate	innate-like (PLZF-high) vs. other	log(odds ratio for innate-like state) per unit increase in TCR-innate	$\beta_{TCR\text{-}innate}$	1.18	[1.15 - 1.21]
Dataset 3	regularized canonical correlation analysis	TCR-innate.rCCA	innate-like (PLZF-high) vs. other	log(odds ratio for innate-like state) per standard deviation increase in TCR score	$\beta_{TCR\text{-}innate.rCCA}$	0.96	[0.94 - 0.98]
Dataset 3	convolutional neural network	TCR-innate.CNN	innate-like (PLZF-high) vs. other	log(odds ratio for innate-like state) per standard deviation increase in TCR score	$\beta_{TCR\text{-}innate.CNN}$	1.14	[1.11 - 1.17]
Dataset 3	regularized logistic regression	TCR-CD8	CD8T vs. CD4T	log(odds ratio for CD8T state) per unit increase in TCR-CD8	$\beta_{TCR\text{-}CD8}$	1.04	[1.02 - 1.06]
Dataset 3	regularized canonical correlation analysis	TCR-CD8.rCCA	CD8T vs. CD4T	log(odds ratio for CD8T state) per unit increase in TCR-CD8	$\beta_{TCR\text{-}CD8.rCCA}$	0.96	[0.95 - 0.98]
Dataset 3	convolutional neural network	TCR-CD8.CNN	CD8T vs. CD4T	log(odds ratio for CD8T state) per unit increase in TCR-CD8	$\beta_{TCR\text{-}CD8.CNN}$	1.08	[1.06 - 1.10]
Dataset 3	regularized logistic regression	TCR-reg	Treg vs. Tconv	log(odds ratio for Treg state) per unit increase in TCR-reg	$\beta_{TCR\text{-}reg}$	0.3	[0.26 - 0.33]
Dataset 3	regularized canonical correlation analysis	TCR-reg.rCCA	Treg vs. Tconv	log(odds ratio for Treg state) per unit increase in TCR-reg	$\beta_{TCR\text{-}reg.rCCA}$	0.22	[0.18-0.26]
Dataset 3	convolutional neural network	TCR-reg.CNN	Treg vs. Tconv	log(odds ratio for Treg state) per unit increase in TCR-reg	$\beta_{TCR\text{-}reg.CNN}$	0.29	[0.26 - 0.33]

Dataset 3	regularized logistic regression	TCR-mem	memory vs. naïve	log(odds ratio for memory state) per unit increase in TCR-mem	$\beta_{TCR\text{-}mem}$	0.14	[0.12-0.15]
Dataset 3	regularized canonical correlation analysis	TCR-mem.rCCA	memory vs. naïve	log(odds ratio for memory state) per unit increase in TCR-mem	$\beta_{TCR\text{-}mem.rCCA}$	0.06	[0.04 - 0.07]
Dataset 3	convolutional neural network	TCR-mem.CNN	memory vs. naïve	log(odds ratio for memory state) per unit increase in TCR-mem	$\beta_{TCR\text{-}mem.CNN}$	0.12	[0.11-0.13]
Dataset 3	regularized logistic regression	TCR-innate	innate-like (PLZF-high) vs. other	area under the receiver-operating curve	AUC	0.84	
Dataset 3	regularized canonical correlation analysis	TCR-innate.rCCA	innate-like (PLZF-high) vs. other	area under the receiver-operating curve	AUC	0.83	
Dataset 3	convolutional neural network	TCR-innate.CNN	innate-like (PLZF-high) vs. other	area under the receiver-operating curve	AUC	0.84	
Dataset 3	regularized logistic regression	TCR-CD8	CD8T vs. CD4T	area under the receiver-operating curve	AUC	0.76	
Dataset 3	regularized canonical correlation analysis	TCR-CD8.rCCA	CD8T vs. CD4T	area under the receiver-operating curve	AUC	0.75	
Dataset 3	convolutional neural network	TCR-CD8.CNN	CD8T vs. CD4T	area under the receiver-operating curve	AUC	0.76	
Dataset 3	regularized logistic regression	TCR-reg	Treg vs. Tconv	area under the receiver-operating curve	AUC	0.58	
Dataset 3	regularized canonical correlation analysis	TCR-reg.rCCA	Treg vs. Tconv	area under the receiver-operating curve	AUC	0.56	
Dataset 3	convolutional neural network	TCR-reg.CNN	Treg vs. Tconv	area under the receiver-operating curve	AUC	0.56	

Dataset 3	regularized logistic regression	TCR-mem	memory vs. naïve	area under the receiver-operating curve	AUC	0.54	
Dataset 3	regularized canonical correlation analysis	TCR-mem.rCCA	memory vs. naïve	area under the receiver-operating curve	AUC	0.52	
Dataset 3	convolutional neural network	TCR-mem.CNN	memory vs. naïve	area under the receiver-operating curve	AUC	0.53	

Table S7. TCR-mem testing within antigen-specific T cell populations, related to Figure 4

Dextramer	number of TCR clones	TCR-mem				Dextramer staining intensity		
		$\beta_{TCR\text{-}mem}$	standard error	two-sided *P* value	one-sided *P* value	β_{stain}	standard error	two-sided *P* value
A0201_KTWGQYWQV_gp100_Cancer	213	0.551458	0.169733063	0.0011582	0.000579	-0.03462	0.157425199	0.8259305
A0201_ELAGIGILTV_MART.1_Cancer	133	0.43441	0.344056071	0.2067276	0.103364	0.104885	0.270631336	0.6983429
B3501_IPSINVHHY_pp65_CMV	189	0.362295	0.173725958	0.0370297	0.018515	0.217297	0.157716691	0.1682752
A0201_KLQCVDLHV_PSA146.154	251	0.359364	0.150101226	0.0166591	0.00833	-0.16454	0.163638581	0.314644
A0201_RMFPNAPYL_WT.1	369	0.260826	0.120435187	0.0303341	0.015167	-0.27559	0.140940083	0.0505426
A0201_SLFNTVATLY_Gag.protein_HIV	289	0.257471	0.137598596	0.0613213	0.030661	-0.1788	0.141611332	0.2067174
B0702_RPHERNGFTVL_pp65_CMV	123	0.240652	0.260966859	0.3564482	0.178224	0.046973	0.241086738	0.845518
A0201_NLVPMVATV_pp65_CMV	285	0.229428	0.123310251	0.0628047	0.031402	0.256855	0.140859045	0.0682292
A0201_YLNDHLEPWI_BCL.X_Cancer	423	0.208398	0.104436734	0.0459943	0.022997	-0.16727	0.109565869	0.1268429
A0201_MLDLQPETT_16E7_HPV	173	0.201871	0.184098771	0.2728451	0.136423	-0.01399	0.180593872	0.9382571
B0702_RPPIFIRRL_EBNA.3A_EBV	292	0.181704	0.165535951	0.2723494	0.136175	0.131365	0.148390503	0.3760118
A0201_KVLEYVIKV_MAGE.A1_Cancer	419	0.160024	0.102307066	0.117782	0.058891	0.100096	0.106599351	0.3477352
A0201_YLLEMLWRL_LMP1_EBV	227	0.146149	0.148382199	0.324648	0.162324	0.227124	0.157172499	0.1484406
A0201_SLLMWITQV_NY.ESO.1_Cancer	179	0.106655	0.157221068	0.4975341	0.248767	-0.33785	0.251830199	0.1797316
A0201_ILKEPVHGV_RT_HIV	221	0.099287	0.144831484	0.4930079	0.246504	-0.08767	0.159934414	0.5835655
A0201_CLGGLLTMV_LMP.2A_EBV	31	0.097583	0.501104777	0.8456001	0.4228	0.169601	0.509334312	0.7391448
A0201_CLLGTYTQDV_Kanamycin.B.dioxygenas	131	0.079872	0.186150629	0.6678705	0.333935	-0.10153	0.188368939	0.5898997
A0201_KVAELVHFL_MAGE.A3_Cancer	189	0.058008	0.164294489	0.7240316	0.362016	0.049257	0.156074147	0.752305
B0702_QPRAPIRPI_EBNA.6_EBV	203	0.032272	0.215240713	0.8808152	0.440408	0.502029	0.168001878	0.002806
A0201_IMDQVPFSV_gp100_Cancer	224	-0.02307	0.158082779	0.8839718	0.558014	-0.30626	0.198812246	0.1234536
A0201_CLLWSFQTSA_Tyrosinase_Cancer	69	-0.065744	0.275968445	0.8117016	0.594149	-0.25564	0.26727697	0.3388423
A0201_FLASKIGRLV_Ca2.indepen.Plip.A2	210	-0.066603	0.137984473	0.6293206	0.68534	0.416214	0.155914783	0.0075965
A0201_GILGFVFTL_Flu.MP_Influenza	641	-0.131149	0.148759387	0.3779842	0.811008	1.639791	0.194482033	3.41E-17
A0201_SLFNTVATL_Gag.protein_HIV	361	-0.149972	0.112261395	0.1815759	0.909212	0.260123	0.126621003	0.0399427
B0702_TPRVTGGGAM_pp65_CMV	165	-0.177197	0.235712103	0.4522004	0.7739	0.509396	0.238236891	0.0325012
A0201_RTLNAWVKV_Gag.protein_HIV	102	-0.184576	0.247608551	0.4560095	0.771995	0.043177	0.230904375	0.851667
A0201_LLFGYPVYV_HTLV.1	281	-0.19268	0.139195973	0.1662864	0.916857	-0.24266	0.13828378	0.0792962
A0201_SLYNTVATLY_Gag.protein_HIV	65	-0.364728	0.349088467	0.2961144	0.851943	0.62293	0.355919602	0.0800839
A0201_LLMGTLGIVC_HPV.16E7_82.91	35	-0.517231	0.603598113	0.391493	0.804253	-0.21958	0.399711203	0.5827607

Figure S1. Unimodal (mRNA) T cell state characterization, related to Figures 1 and 2

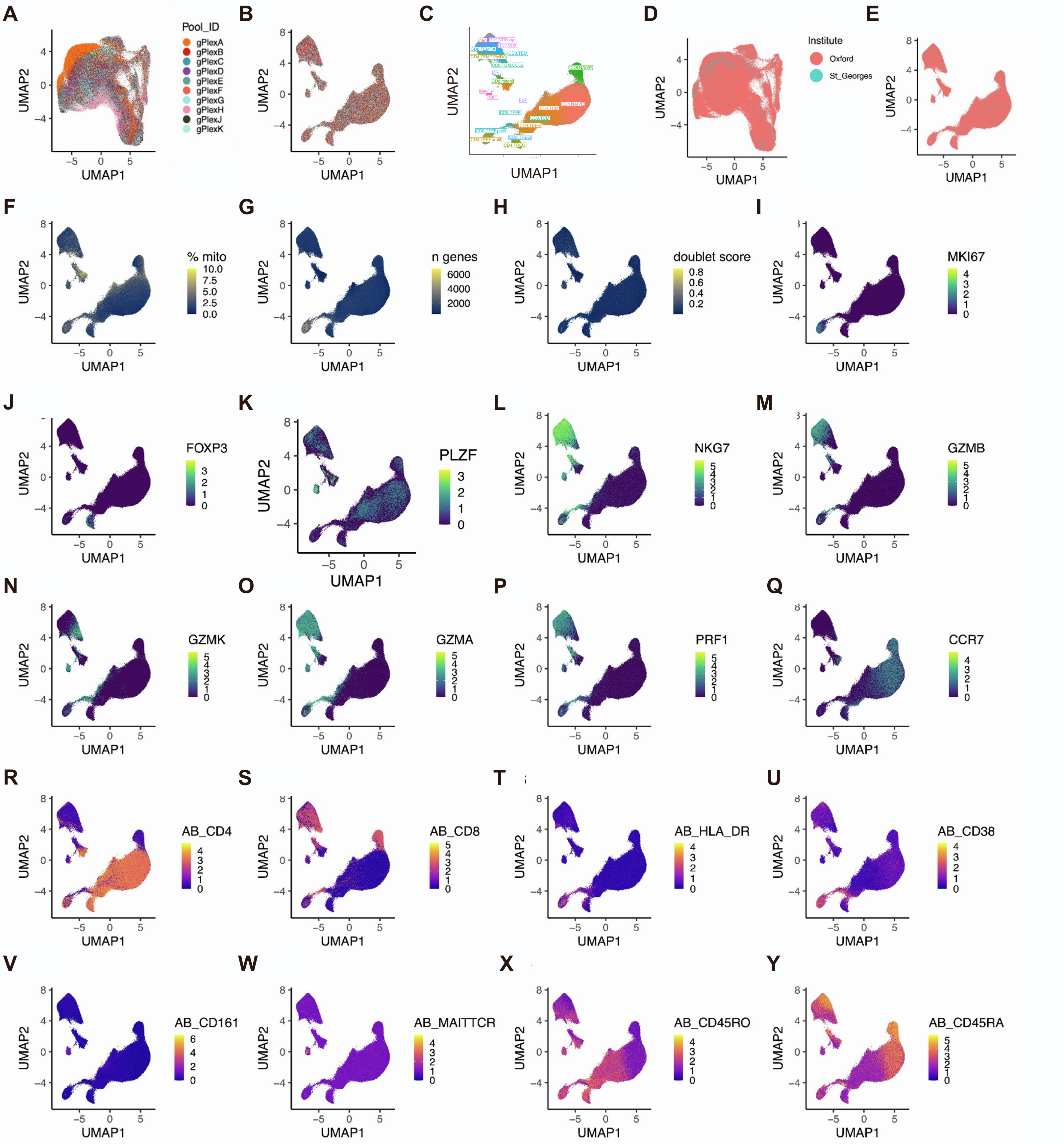

Figure S1. (A) UMAP of Dataset 1 T cells based on the first 20 principal components (PCs) of gene expression, prior to batch correction. Pool_ID indicates sequencing library plex (Fluidigm Cell-ID 20-Plex Pd Barcoding Kit). **(B)** Dataset 1 T cells colored as in (A), now arranged in a UMAP following batch-correction by Harmony. **(C)** Our UMAP of Dataset 1 T cells based on the first 20 principal components (PCs) of gene expression, following batch-correction by Harmony. T cells are colored by the "Annotation_minor_subset" label assigned by the original authors. **(D)** Dataset 1 T cells prior to batch correction, colored by hospital recruitment site. **(E)** Dataset 1 T cells following batch correction, colored by hospital recruitment site. **(F)** Dataset 1 T cells colored by percentage of UMIs aligned to mitochondrial transcripts. **(G)** Dataset 1 T cells colored by the number of unique genes with nonzero counts. **(H)** Dataset 1 T cells colored by Scrublet doublet scores. **(I) - (Q)** Dataset 1 T cells colored by log(CP10K + 1) normalized expression of marker transcripts. **(R) - (Y)** Dataset 1 T cells colored by centered-log-ratio (CLR) normalized TotalSeq UMIs.

Figure S2. Multimodal (CITE-seq) T cell state characterization, related to Figures 2 and 3

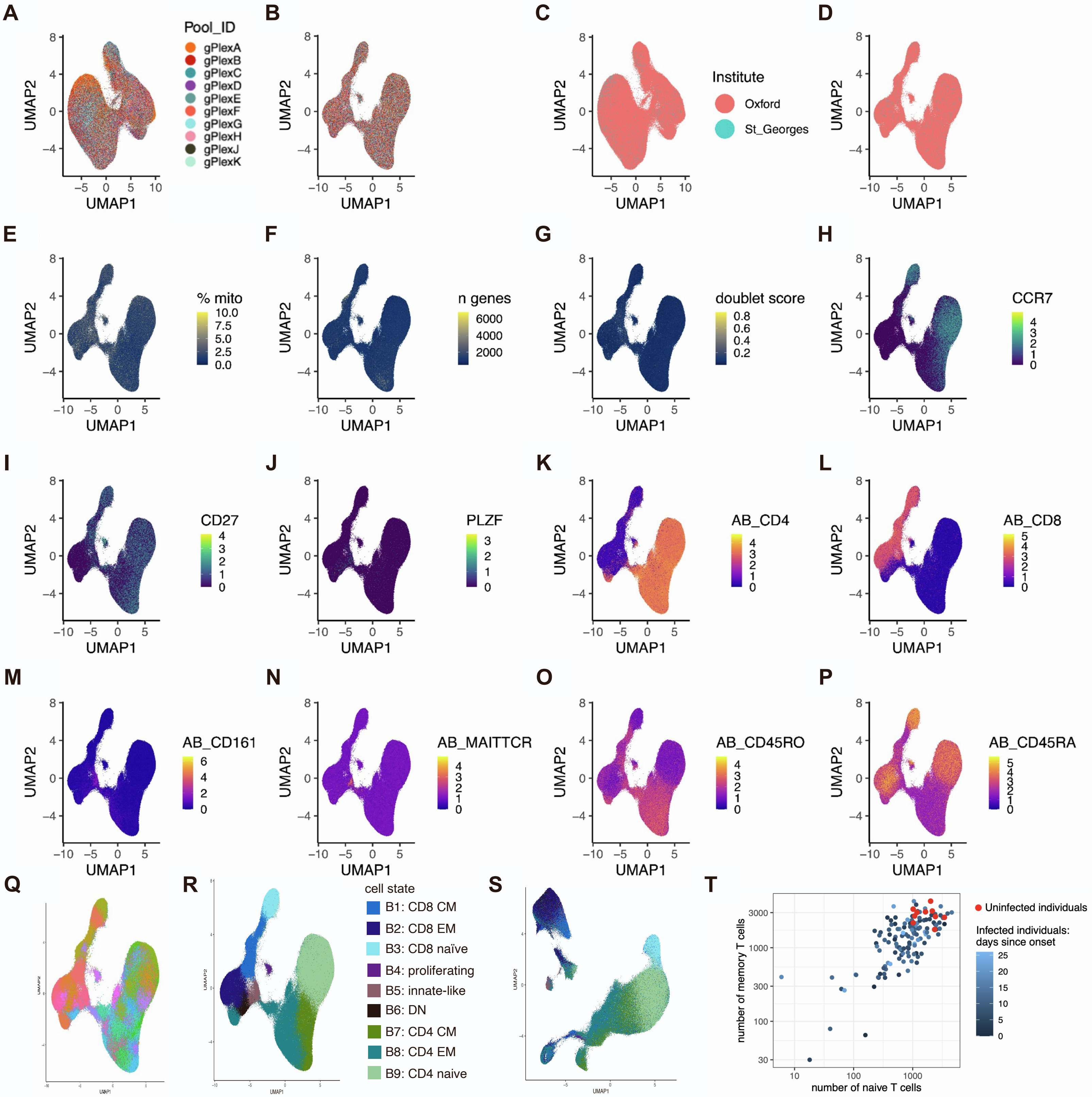

Figure S2. (A) UMAP of Dataset 1 T cells, based on the first 10 gene expression-based canonical variates from canonical correlation analysis (CCA) applied to the scaled and normalized expression of 4423 variable genes and 10 surface proteins (Supplementary Table 1) relevant to CD4, CD8, central memory (CM), and effector memory (EM) distinctions (Methods). Pool_ID indicates sequencing library plex (Fluidigm Cell-ID 20-Plex Pd Barcoding Kit). **(B)** Dataset 1 T cells colored as in (A), following batch-correction by Harmony. **(C)** UMAP as in (A), colored by hospital recruitment site. **(D)** UMAP as in (B), colored by hospital recruitment site. **(E)** Dataset 1 T cells colored by percentage of UMIs aligned to mitochondrial transcripts. **(F)** Dataset 1 T cells colored by the number of unique genes with nonzero counts. **(G)** Dataset 1 T cells colored by Scrublet doublet scores. **(H) - (J)** Dataset 1 T cells colored by log(CP10K + 1) normalized UMI counts of marker transcripts. **(K) - (P)** Dataset 1 T cells colored by centered-log-ratio (CLR) normalized expression of TotalSeq UMIs. **(Q)** Dataset 1 T cells assigned to 60 discrete clusters by Louvain clustering, implemented via Seurat::RunModularityClustering at resolution 4.0. **(R)** 60 Louvain clusters from (q) collapsed into 9 T cell state annotations (B1-B9). **(S)** Dataset 1 T cells colored by annotations B1-B9, rearranged into their original protein-agnostic UMAP (Supplementary Figure 1a). **(T)** Scatterplot of individuals in Dataset 1, comparing the number of naive T cells (x axis) to the number of memory T cells (y axis) in each individual's sample. Each point is colored by the individual's infection status.

Figure S3, Canonical variate interpretation, related to Figure 2

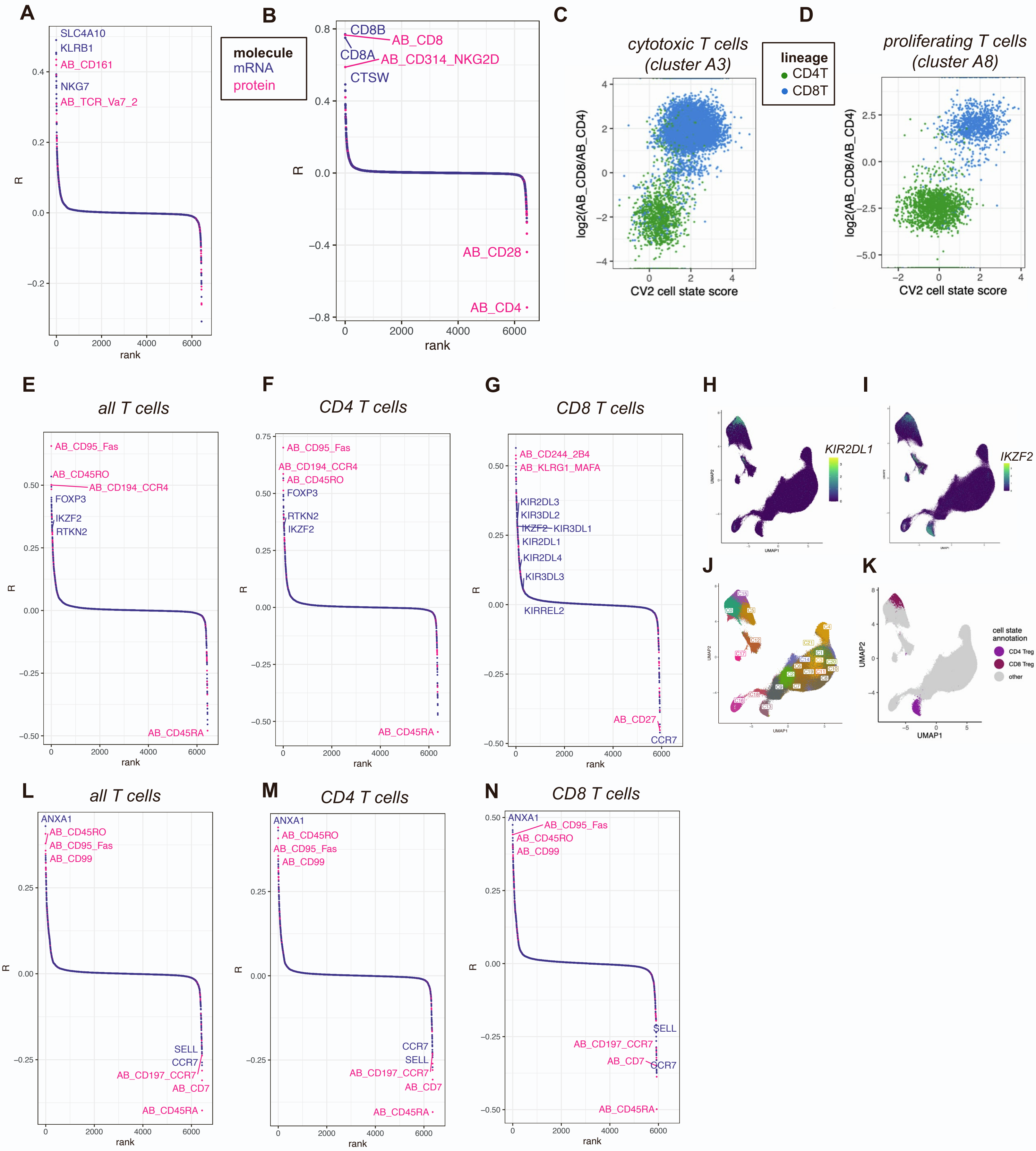

Figure S3. **(A)** Correlations between CV1 T cell state score and expression of each variable gene (purple) as well as each TotalSeq surface protein (pink) in Dataset 1. **(B)** Correlations between CV2 T cell state score and expression of each variable gene (purple) as well as each TotalSeq surface protein (pink) in Dataset 1. **(C)** Among cytotoxic T cells, the CV2 cell state score delineates CD4 and CD8 populations. **(D)** Among proliferating T cells, the CV2 cell state score delineates CD4 and CD8 populations. **(E)** Correlations between CV3 T cell state score and expression of each variable gene (purple) as well as each TotalSeq surface protein (pink) in Dataset 1. **(F)** Correlations as in (E), restricted to CD4 T cells. **(G)** Correlations as in (E), restricted to CD8 T cells. **(H-I)** UMAP of Dataset 1 T cells, colored by log(CP10K +1) normalized expression of *KIR2DL1* and *IKZF2*, respectively. **(J)** Louvain clustering of Dataset 1 T cells at resolution 2.0. **(K)** Cell annotations with respect to Treg state for Dataset 1 T cells. **(L)** Correlations between CV4 T cell state score and expression of each variable gene (purple) as well as each TotalSeq surface protein (pink) in Dataset 1. **(M)** Correlations as in (L), restricted to CD4 T cells. **(N)** Correlations as in (L), restricted to CD8 T cells

CV = Canonical Variate

Figure S4, Generalizability of TCR scoring functions, related to Figure 2 and Figure 3

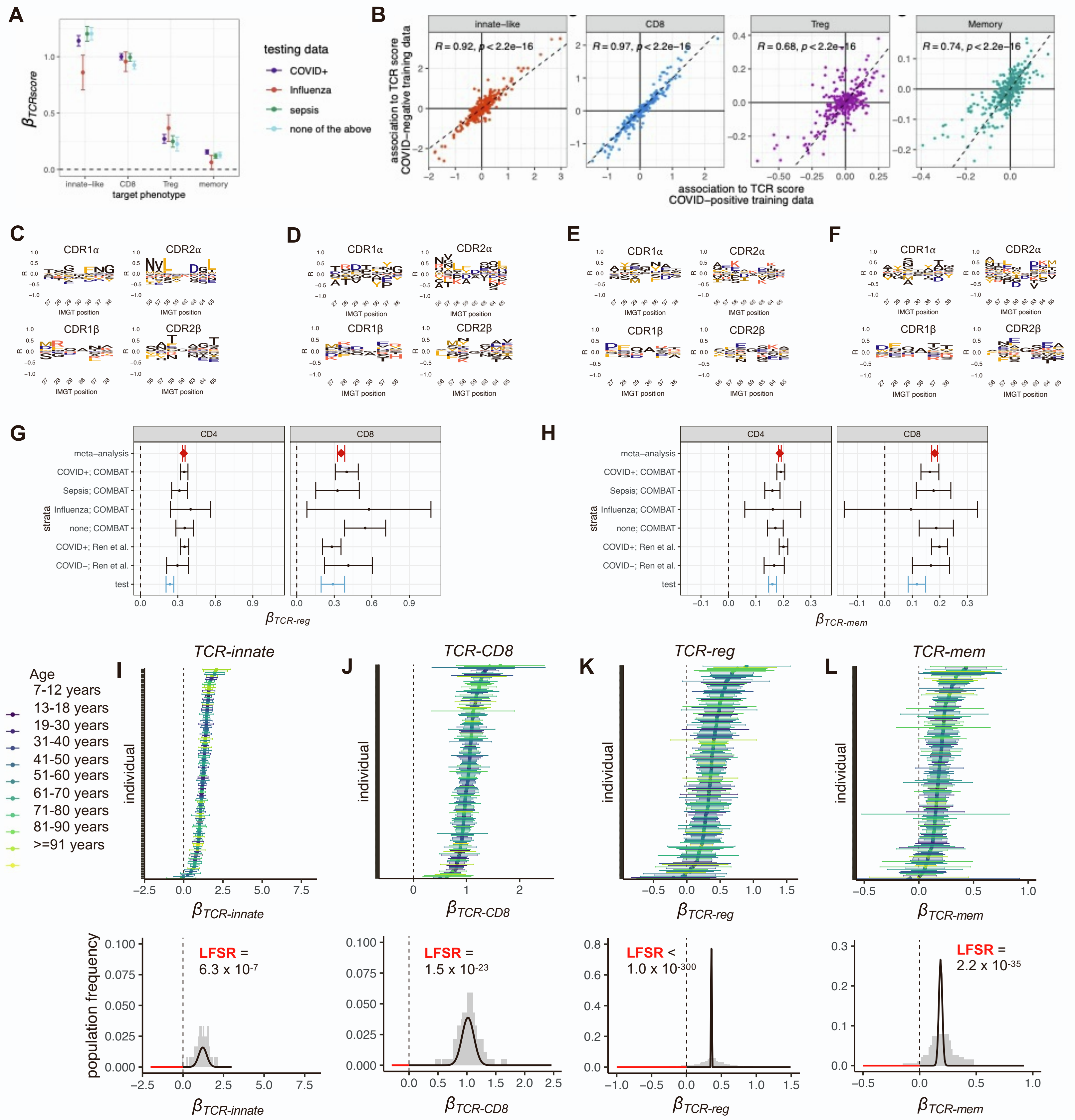

Figure S4. **(A)** Effect sizes between TCR scores and target T cell phenotypes, measured as $\beta_{TCRscore}$, with 95% confidence interval. TCR scores were scaled to have mean 0 and variance 1 in the training dataset. Effect sizes and standard errors were computed via mixed-effects logistic regression. In this analysis, all TCR scoring functions were trained on only COVID-positive samples. We then applied these new TCR scoring functions to four testing datasets of held-out observations: COVID+ (purple), Influenza (red), Sepsis (green), and none of the above (blue), all from Dataset 1. **(B)** Scatterplot of 319 TCR sequence features and their association to the given TCR score when trained on COVID-positive data (x axis) compared to COVID-negative data (y axis). Here, each TCR sequence feature is a combination of IMGT position and amino acid identity, across CDR1-3 of both the alpha and beta chains. For reliable estimates, we only include amino acids with frequency >= 0.05 at the given position. Plotted association values are coefficient estimates from linear regression with TCR score as the response variable. *P* values are computed by t tests on the Pearson's product moment correlation coefficient, n = 319 TCR sequence features. **(C)** TCR features for TCR-innate in CDR1 and CDR2 regions, visualized as Bonferroni-significant marginal Pearson correlations to each amino acid. **(D)** TCR features for TCR-CD8, computed as in (C) **(E)** TCR features for TCR-reg, computed as in (C) **(F)** TCR features for TCR-mem, computed as in (C). **(G)** Forest plot depicting the association between TCR-reg and Treg state for T cells from each subset of individuals, further stratified by CD4 lineage (left) and CD8 lineage (right). CD4 lineage and CD8 lineage are designated via clusters B1-B9 **(H)** Forest plot depicting association between TCR-mem and memory state for T cells stratified as in (G). Meta-analytic $\beta_{TCR\text{-}reg}$ and $\beta_{TCR\text{-}mem}$ are estimated by fixed-effects inverse-variance-weighted meta-analysis, applied to the 6 training subsets. **(I)** Top: forest plot shows 95% CIs for $\beta_{TCR\text{-}innate}$ for Dataset 1 and Dataset 2 T cells stratified by individual. $\beta_{TCR\text{-}innate}$ estimates are computed via logistic regression for innate-like transcriptional fate, done separately in each individual. Bottom: histogram shows the same $\beta_{TCR\text{-}innate}$ estimates in gray, with the estimated distribution of $\beta_{TCR\text{-}innate}$ overlaid in black. Parameters for the distribution of $\beta_{TCR\text{-}innate}$ are estimated via random effects meta-analysis. Red-shaded area corresponds to the local false sign rate (LFSR), the estimated proportion of individuals for whom TCR-innate does not demonstrate a positively signed association to innate-like transcriptional fate. **(J)** Analogous to (I), for TCR-CD8 and CD8 T cell fate. **(K)** Analogous to (I), for TCR-reg and Treg cell fate. **(L)** Analogous to (I), for TCR-mem and memory T cell state.

Figure S5, Machine learning model comparison, related to Figure 2 and Figure 3

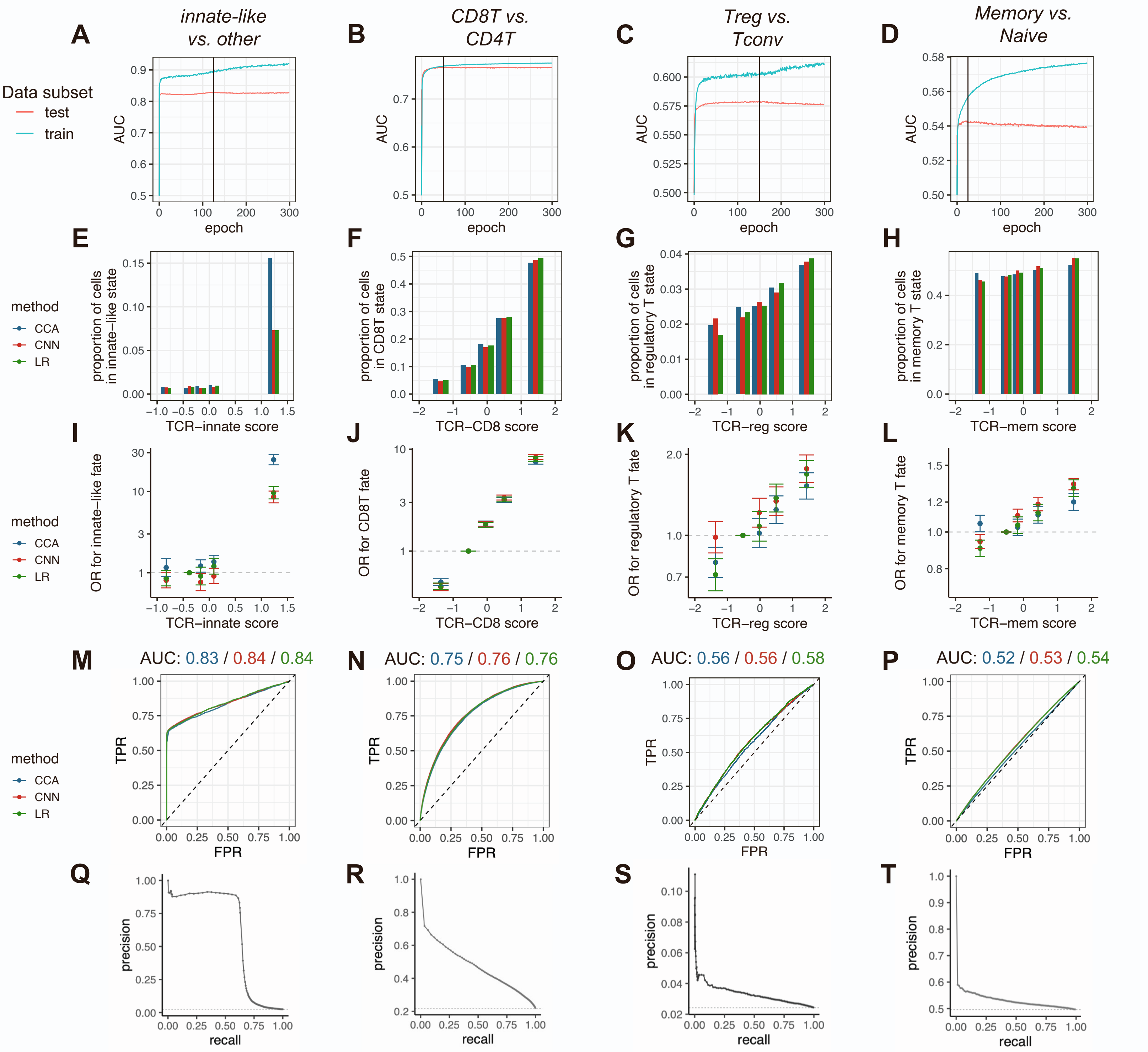

Figure S5. With Dataset 3 as an external validation cohort, we compared TCR scoring functions from rCCA (blue), ridge-regularized logistic regression (green), and Convolutional Neural Networks (CNN, red). All three scoring functions were trained on the same observations (70% of clones from Dataset 1 and Dataset 2). For both training and testing data, expanded T cell clones were de-duplicated by picking one representative cell at random. **(A-D)** At each epoch of Convolutional Neural Network training, we calculated the area under the receiver operating statistic curve (AUC) in the training and testing subsets. To avoid overfitting to the training data, we used the fitted model marked by the vertical line. **(E)** Each bar represents a decile of TCR score computed by one of the methods. Y-axis denotes the proportion of T cell clones from Dataset 3 observed in the innate-like (*PLZF*^{high}, cluster A9) T cell state. **(F-H)** Analogous to (E), for CD8 T cell fate, Treg fate, and memory fate, respectively. **(I)** Each point represents a decile of TCR score computed by one of the methods. We compute the odds ratio (OR, y-axis) for the innate-like (*PLZF*^{high}, cluster A9) T cell state for T cells in each decile compared to T cells in the fifth decile. 95% CIs (error bars) and *P* values computed via mixed-effects logistic regression. **(J-L)** Analogous to (I), for CD8 T cell fate, Treg fate, and memory fate, respectively. **(M)** Receiver operating characteristic (ROC) curve for TCR-based classifiers of innate-like T cell fate. **(N-P)** Analogous to (m), for CD8 T cell fate, Treg fate, and memory fate, respectively. **(Q)** Precision-recall curve for TCR-innate as a classifier for innate-like T cell fate. **(R)** Precision-recall curve for TCR-CD8 as a classifier for CD8 T cell fate. **(S)** Precision-recall curve for TCR-reg as a classifier for Treg cell fate. (**T**) Precision-recall curve for TCR-mem as a classifier for memory versus naïve state.

Figure S6, TCR transduction experiments, related to Figure 4

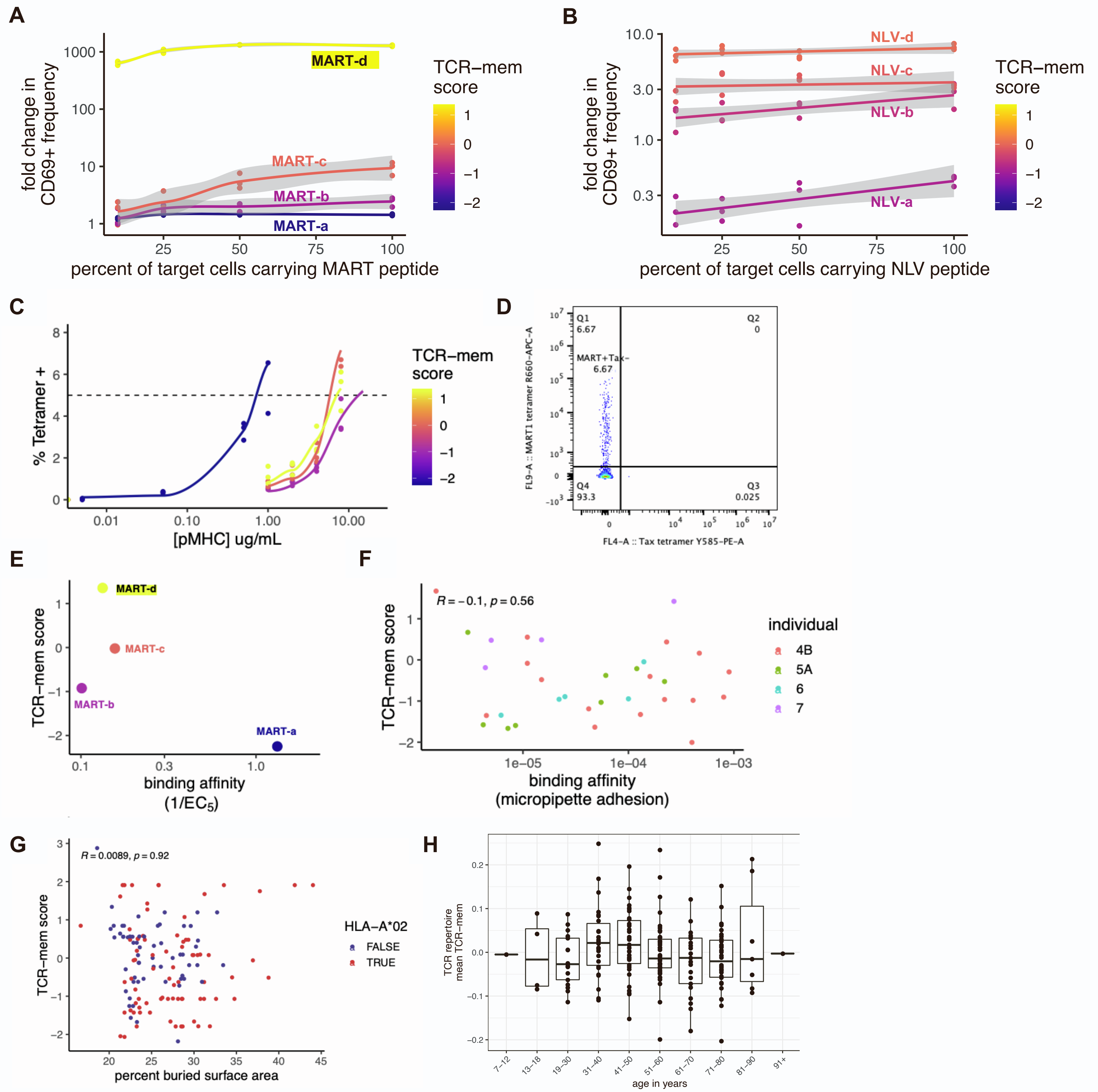

Figure S6. (A) Dose-response curves for Jurkat activation with respect to increasing concentrations of antigen presenting cells (APCs) that express the MART-1 antigen. Activation is measured by fold change in the frequency of CD69+ Jurkat cells compared to background, in which 0% of APCs express the MART-1 antigen. **(B)** Same as (A), with NLV- reactive TCRs and the NLV- antigen. **(C)** Percent of Jurkat cells staining positively for MART-1 tetramer across a range of tetramer concentrations, for each TCR-transduced population. For (A-C), data points denote measurements (in triplicate); curves denote fitted dose-response curves. **(D)** Representative flow cytometry plot, depicting staining for the MART-1 tetramer (y-axis) compared to negative control Tax tetramer (x-axis) for Jurkat cells expressing the MART-c TCR sequence. **(E)** Scatterplot comparing TCR-mem score to MART-1 binding affinity (1/EC_5, estimated with a four-parameter log logistic function through R package drc). **(F)** Antigen binding affinity measured via micropipette adhesion (x-axis) in an external study compared to TCR-mem score (y-axis) for 33 TCRs that bind the hepatitis C virus (HCV) antigen KLVALGINAV on HLA-A*02. Color indicates the study ID of the HCV-seronegative individual from which each TCR was sampled. *P* value is computed by a t test on the Pearson's product moment correlation coefficient, n = 33 TCRs. **(G)** Scatterplot of 138 TCR-pMHC crystal structures from the Protein Data Bank (PDB), comparing TCR-mem score (y-axis) to the surface area buried between TCR and pMHC (x-axis). Buried surface areas are estimated through PDBePISA. *P* value is computed by a t test on the Pearson's product moment correlation coefficient, n = 138 structures. **(H)** Mean TCR-mem score for each individual's TCR repertoire sample (y-axis), plotted against the individual's age bracket (x-axis). Data consists of training observations from Dataset 1 and Dataset 2.

Figure S7, Dextramer analysis, related to Figure 4

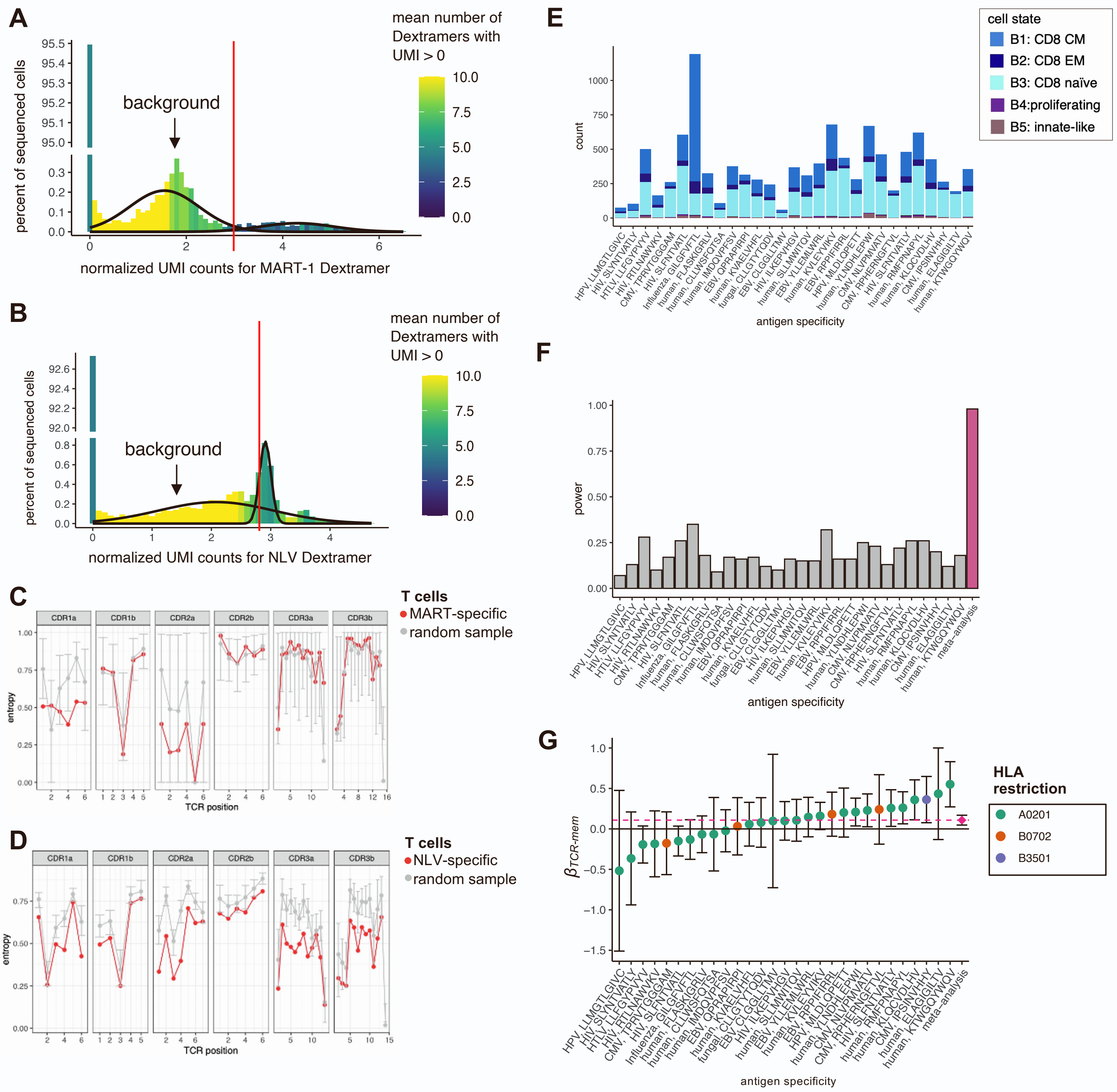

Figure S7. (A-B) Normalized Dextramer UMI counts and demarcation of background staining for two representative Dextramers, **(A)** MART-1 and **(B)** NLV. UMI counts were normalized by a custom negative binomial regression model, see Methods. Black outlines denote the distributions inferred by a Gaussian mixture model (two components, R package "mclust" v5.4.8). Red vertical lines mark the gates we set to delineate background staining from T cells specific for the given Dextramer, following careful visual inspection. **(C)** TCR sequence conservation among T cells inferred to recognize the MART-1 antigen (red), compared to 1000 random resamples of a matched number of cells from Dataset 5 (gray). The point denotes the mean; the error bar denotes the minimum and maximum entropies observed in the 1000 random resamples. (**D**) Analogous to (C), for the NLV antigen. **(E)** Cell counts among each of the antigen-specific populations in Dataset 5. **(F)** Statistical power to detect $\beta_{TCR\text{-}mem}$ within each Dextramer-specific population. For each Dextramer *j* recognized by n_j cells, we sample n_j cells from Dataset 3, in which $\beta_{TCR\text{-}mem}$ is estimated to be 0.13. We use mixed-effects logistic regression to re-estimate $\beta_{TCR\text{-}mem}$ in this sample of size n_j. We repeat this process 100 times, for each Dextramer. On each iteration, we also conduct random effects meta-analysis across Dextramers (shown in pink). **(G)** TCR-mem effect size ($\beta_{TCR\text{-}mem}$) within each antigen-specific population, quantified as the natural logarithm of the odds ratio for memory vs. naive state per unit increase in TCR-mem. Dashed pink line denotes meta-analytic $\beta_{TCR\text{-}mem}$. Meta-analytic $\beta_{TCR\text{-}mem}$, 95% CI, and *P* value are computed via random-effects meta-analysis.
